# Global intercomparison of polyurethane foam passive air samplers evaluating sources of variability in SVOC measurements

**DOI:** 10.1016/j.envsci.2021.08.003

**Published:** 2021-11

**Authors:** Lisa Melymuk, Pernilla Bohlin Nizzetto, Tom Harner, Kevin B. White, Xianyu Wang, Maria Yumiko Tominaga, Jun He, Jun Li, Jianmin Ma, Wan-Li Ma, Beatriz H. Aristizábal, Annekatrin Dreyer, Begoña Jiménez, Juan Muñoz-Arnanz, Mustafa Odabasi, Yetkin Dumanoglu, Baris Yaman, Carola Graf, Andrew Sweetman, Jana Klánová

**Affiliations:** a RECETOX, Masaryk University, Brno, Czech Republic; b NILU - Norwegian Institute for Air Research, Kjeller, Norway; c Air Quality Processes Research Section, Environment and Climate Change Canada, Toronto, Canada; d Queensland Alliance for Environmental Health Sciences (QAEHS), The University of Queensland, Australia; e CETESB - São Paulo State Environmental Company, São Paulo, Brazil; f Department of Chemical and Environmental Engineering, University of Nottingham Ningbo China, Ningbo, China; g State Key Laboratory of Organic Geochemistry, Guangzhou Institute of Geochemistry, Chinese Academy of Sciences, Guangzhou, China; h College of Urban and Environmental Sciences, Peking University, Beijing, China; i International Joint Research Center for Persistent Toxic Substances (IJRC-PTS), Harbin Institute of Technology, Harbin, China; j Hydraulic Engineering and Environmental Research Group (GTAIHA), Universidad Nacional de Colombia, Manizales, Colombia; k Eurofins GfA GmbH (Now Operating Under the Name ANECO Institut für Umweltschutz GmbH & Co), Germany; l Department of Instrumental Analysis and Environmental Chemistry, IQOG-CSIC, Madrid, Spain; m Department of Environmental Engineering, Dokuz Eylul University, Buca-Izmir, Turkey; n Lancaster Environment Centre, Lancaster University, UK

**Keywords:** Passive air sampling, Global air monitoring, Persistent organic pollutants, Semi-volatile organic compounds, Stockholm Convention, PUF disk

## Abstract

Polyurethane foam passive air samplers (PUF-PAS) are the most common type of passive air sampler used for a range of semi-volatile organic compounds (SVOCs), including regulated persistent organic pollutants (POPs) and polycyclic aromatic hydrocarbons (PAHs), and emerging contaminants (e.g., novel flame retardants, phthalates, current-use pesticides). Data from PUF-PAS are key indicators of effectiveness of global regulatory actions on SVOCs, such as the Global Monitoring Plan of the Stockholm Convention on Persistent Organic Pollutants. While most PUF-PAS use similar double-dome metal shielding, there is no standardized dome size, shape, or deployment configuration, with many different PUF-PAS designs used in regional and global monitoring. Yet, no information is available on the comparability of data from studies using different PUF-PAS designs. We brought together 12 types of PUF-PAS used by different research groups around the world and deployed them in a multi-part intercomparison to evaluate the variability in reported concentrations introduced by different elements of PAS monitoring. PUF-PAS were deployed for 3 months in outdoor air in Kjeller, Norway in 2015–2016 in three phases to capture (1) the influence of sampler design on data comparability, (2) the influence of analytical variability when samplers are analyzed at different laboratories, and (3) the overall variability in global monitoring data introduced by differences in sampler configurations and analytical methods. Results indicate that while differences in sampler design (in particular, the spacing between the upper and lower sampler bowls) account for up to 50 % differences in masses collected by samplers, the variability introduced by analysis in different laboratories far exceeds this amount, resulting in differences spanning orders of magnitude for POPs and PAHs. The high level of variability due to analysis in different laboratories indicates that current SVOC air sampling data (i.e., not just for PUF-PAS but likely also for active air sampling) are not directly comparable between laboratories/monitoring programs. To support on-going efforts to mobilize more SVOC data to contribute to effectiveness evaluation, intercalibration exercises to account for uncertainties in air sampling, repeated at regular intervals, must be established to ensure analytical comparability and avoid biases in global-scale assessments of SVOCs in air caused by differences in laboratory performance.

## Highlights

• 12 types of PUF passive air samplers were deployed in an intercomparison exercise.
• Differences in sampler design lead to small variations in sampler performance.
• Replicate PUF air samples were sent to 14 laboratories to assess variability in data.
• Laboratories report very different concentrations of POPs, PAHs in identical samples.
• Analytical uncertainties must be addressed for comparability of air monitoring data.

## 1 Introduction

Long-term global data on atmospheric levels of semi-volatile organic compounds (SVOCs), including polycyclic aromatic hydrocarbons (PAHs) and persistent organic pollutants (POPs), such as polychlorinated biphenyls (PCBs), organochlorine pesticides (OCPs), and polybrominated diphenyl ethers (PBDEs), are a fundamental need in efforts to reduce emissions and minimize human and environmental exposure. This need has been formalized in the requirements of international actions, such as the Stockholm Convention on POPs (Articles 11 and 16) implemented through the Global Monitoring Plan (GMP), the UNECE Convention on Long-Range Transboundary Air Pollution (CLRTAP), and the development of a Global Earth Observation System of Systems (GEOSS) to increase our understanding of global processes and to underpin decision-making through sharing of accessible, high quality interoperable environmental data.

The GMP has a clear policy mandate to collect comparable, harmonized and reliable information on POP levels in core environmental matrices, one of which is ambient air. The Global Observation System for Persistent Organic Pollutants (GOS4POPs) is an initiative within the Group on Earth Observations (GEO) to increase the availability and quality of Earth observation data on POPs, and improve data availability and interoperability across POP monitoring networks, providing support for international conventions on toxic compounds (Stockholm Convention, CLRTAP) and on-going international programs (e.g., GMP, European Monitoring and Evaluation Programme (EMEP)).

Polyurethane foam passive air samplers (PUF-PAS) are widely used in international air monitoring of POPs (Borůvková et al., 2015; Muñoz-Arnanz et al., 2016; Pozo et al., 2009; Wania and Shunthirasingham, 2020) and other semi-volatile organic compounds (SVOCs). The spatial coverage and ease-of-use of PUF-PAS has been crucial in enabling the development of international air monitoring programs such as GAPS (Global Atmospheric Passive Sampling) and MONET (Lee et al., 2007; Muñoz-Arnanz et al., 2018, 2016; Pozo et al., 2006; Přibylová et al., 2012; Rauert et al., 2018; Roscales et al., 2018a; White et al., 2021), and their use in many individual case studies has greatly increased our knowledge of atmospheric levels of SVOCs. Following the entry-into-force of the Stockholm Convention in 2004, the GMP was established to secure monitoring data in core media (ambient air, breast milk, human blood) and became a strong driver for the development of passive air sampling programs to address global data gaps, especially given the simplicity and relatively low cost of passive air samplers (Klánová and Harner, 2013). The first GMP Report (UNEP, 2009) called for improved collaboration within and among regions, and establishment of strategic partnerships with expert laboratories and programs to address the challenges in setting up new POP monitoring programs that can continually adapt to include newly listed POPs. To mobilise such data and ensure their interoperability, we must move towards more harmonised monitoring frameworks with comprehensive datasets. While internal consistency of data within individual programs is necessary to assess long-term trends, the comparability of data among different programs must also be improved so that datasets can be combined for more effective global assessment. However, despite the intended goal of global-scale comparability, differences in analytical methods and sampler configurations between institutes and monitoring programs may affect performance (Holt et al., 2017; Markovic et al., 2015; Roscales et al., 2018b) and decrease the comparability of international monitoring data (Su and Hung, 2010).

The simple design of the PUF-PAS has led to many individually-designed versions around the globe, all following the same original PUF-PAS concept (Shoeib and Harner, 2002) of a PUF disk protected by a metal double-dome housing, but without standardized geometry. In a previous comparison of three samplers, differences in sampler design were found to have no discernable effect on PUF-PAS uptake rates (Chaemfa et al., 2008), however, today the use of PUF-PAS has greatly expanded due to ease of deployment and use, and sampler designs differ to a much greater extent. At least 15 different designs are regularly used, with differences in dome size and shape, placement of the PUF disk relative to the gap between domes, size and density of the PUF disk itself, and deployment practices (i.e., fixed versus freely hanging). In addition, there are clear differences across laboratories in analytical methodology applied to PUF processing and SVOC analysis, which have the potential to lead to large variabilities in reported concentrations (Su et al., 2011; Su and Hung, 2010). Current efforts to harmonize and synthesize global SVOC monitoring combine data collected from different PUF-PAS sampler designs and analyzed in different laboratories (e.g., GMP incorporates PUF-PAS data from five different air monitoring networks globally; Fig. S1), but lack information on how the variability introduced by physical PUF-PAS design parameters compares to the analytical variability between laboratories.

To evaluate the comparability of global SVOC data, we established an international intercomparison in 2015 to evaluate sources of variability in PUF-PAS-generated data. Institutes from 12 countries (Australia, Brazil, Canada, China, Colombia, Czechia, Germany, Mexico, Norway, Spain, Turkey, UK) participated in the intercomparison, covering many of the major research groups using PUF-PAS and including most of the monitoring networks/laboratories that have reported PUF-PAS data to the Stockholm Convention GMP Data Warehouse for the 3^rd^ Global Monitoring Report on Persistent Organic Pollutants. We note that such an exercise is only possible due to the simplicity and small size of the PUF-PAS samplers, whereas a similar effort for active air samplers would not be feasible for logistical reasons. The PUF-PAS intercomparison consisted of three phases to address the following questions:

o what is the variability introduced by differences in PUF-PAS sampler designs and deployment practices? (Phase 1)
o what is the variability introduced by differences in analytical methods/performance between laboratories? (Phase 2)
o what is the overall variability/comparability between PUF-PAS-derived air concentrations for POPs from different programs/laboratories? (Phase 3)

This study evaluates the variability in SVOC measurements across these three phases due to differences in sampler design and laboratory performance to assess the comparability of reported SVOC monitoring data from PUF-PAS across the globe.

## 2 Methods

**Fig. 1:**
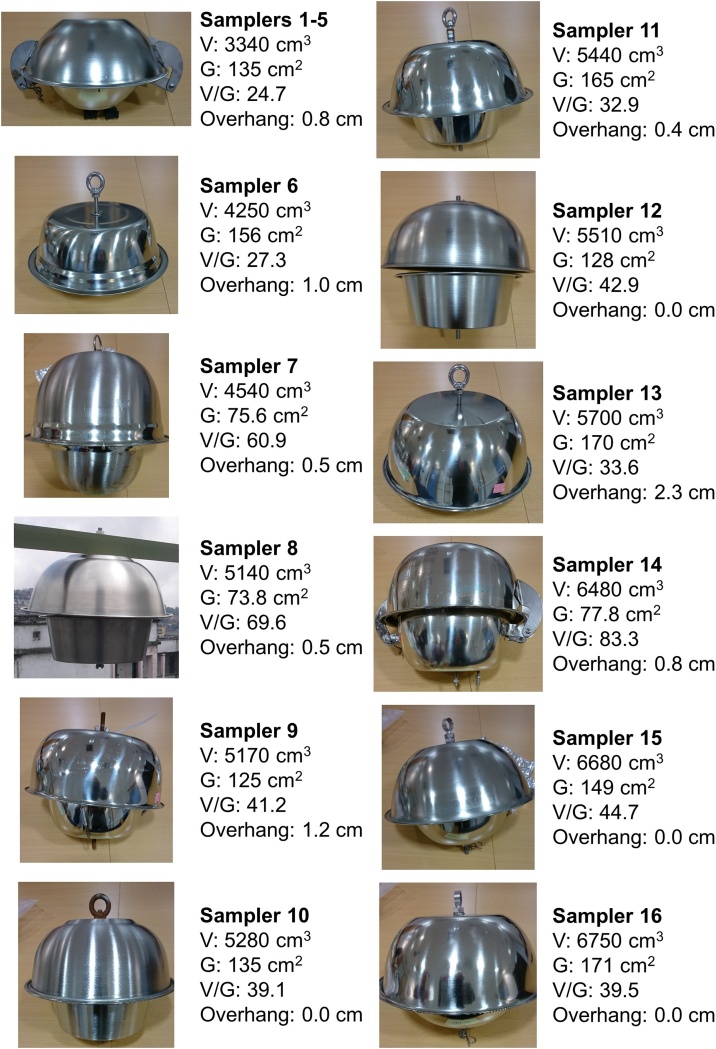
Sampler designs used in the study and their basic dimensions. V indicates volume, G indicates the area of the horizontal gap between upper and lower dome, V/G is the ratio of the dome volume to gap area, and overhang is the distance the upper dome extends over the lower dome.

Laboratory groups known to routinely use PUF-PAS to quantify SVOCs in air were contacted and invited to join the study. In all, the study included 15 participating research institutes (Table S1) using 12 different PUF-PAS sampler designs (Fig. 1). The institutes supplied their own PUF disks and PAS housings. PUF-PAS designs differed by housing dimensions (dome shape, internal volume, overhang, gap diameter) and/or type of PUF disk (details of the individual designs are given in Table S2). All equipment was kept separated under strict regimes. The PUF disks and housings were pre-cleaned at the Norwegian Institute for Air Research (NILU) before deployment in each phase of the study. All samples in the intercomparison study were deployed at the same site, located in Kjeller, outside Oslo, Norway. The site is semi-rural near grass fields, with a mix of residential, and office buildings at a short distance. Meteorological parameters corresponding with the deployment periods of each study phase are given in Tables S3 and S4. The study was divided into three phases, each addressing a key aspect of monitoring data comparability, as described below.

### 2.1 Phase 1 – different samplers, same PUFs, same laboratory

The objective of Phase 1 was to isolate and identify the specific influence of different PAS housings on sampler uptake. For Phase 1, 16 passive air samplers (consisting of 12 different designs) were collected from the 15 participating laboratories. Each laboratory’s PAS design (different housing and installation parameters, but with identical PUF disks) were deployed simultaneously at the field site in Kjeller, Norway for 80 days from April 1, 2016 to June 13, 2016. Samplers were deployed along a 50 m section of a wire fence at a height of 2 m (Fig S2). This therefore addressed differences in both sampler design and installation parameters, including fixed rigid installations for some samplers and free-swinging installations for others, following the method of the participating laboratory. The PUF disks had a density of 2.70 × 10^4^ g/m^3^, mass of 5.9 g, diameter of 14.1 cm, and thickness of 1.4 cm. Average daily ambient temperature during Phase 1 deployment was 9.2 °C (range −4.6 to +28.5 °C) and average wind speed was 2.8 m/s (range 1.3–5.6 m/s) (Table S3). After 80 days, the PUF-PAS were collected, and PUF disks and three field blanks were packed individually in pre-cleaned aluminum foil and shipped to the Trace Analytical Laboratories of RECETOX, Czechia for SVOC analysis.

All PUF disks were analyzed according to accredited analytical methods (ČSN EN ISO 17025: 2018) for 8 PCB congeners (7 indicator PCBs + PCB 11), 12 OCPs (chlorobenzenes, hexachlorocyclohexanes - HCHs, dichlorodiphenyltrichloroethane and associated metabolites - DDX compounds), 29 PAHs and 10 PBDE congeners; compounds are listed in Table S5. Full details on the analytical methods used by RECETOX can be found in Kalina et al. (2017). Recoveries were tracked using deuterated PAHs (d8-naphthalene, d10-phenanthrene, d12-perylene) and non-environmental PCBs (PCB 30, PCB 185) (Table S6). PAH, PCB and OCP masses were adjusted for recoveries based on the closest corresponding recovery standard. PBDEs were quantified by isotope dilution. Method detection limits (MDLs) were determined based on the field blanks; MDL=[avg. mass in field blanks]+3*[standard dev. of field blanks] (Table S7). If a compound was below detection in all field blanks, the instrumental detection limit was taken as the MDL. All results are reported as mass per PUF disk without conversion to air concentration.

### 2.2 Phase 2 – same samplers, same PUFs, different laboratories

The objective of Phase 2 was to identify purely analytical variability between laboratories. Fourteen identical PUF-PAS samplers (Sampler 15 from Fig. 1) were deployed at the Norwegian field site (deployment height 2 m) for 81 days from September 11, 2015 to December 1, 2015. Average daily ambient temperature during deployment was 5.9 °C (range -12.0 to +19.6 °C) and the average wind speed was 1.9 m/s (range 0–4.9 m/s) (Table S4). After 81 days each PUF disk and a corresponding field blank were collected, wrapped in pre-cleaned aluminum foil and sealed in plastic zip-top bags, packed in a padded envelope and sent to the 15 participating laboratories. Participating laboratories were asked to analyze the PUFs according to their in-house methods and report masses for seven PCB congeners, 10 OCPs, 10 PBDE congeners, and 16 PAHs to an Excel template. All results were reported as mass per PUF disk without conversion to air concentration. Details of the individual methods for each laboratory are given in Table S8. Most laboratories used Soxhlet extraction while three laboratories used accelerated solvent extraction (ASE) and one used a Büchi system. Seven different solvent combinations were used, while only three different clean-up methods were used. Not all laboratories reported all sets of compounds, resulting in data for PCBs from 11 laboratories, OCPs from 11 laboratories, PBDEs from 10 laboratories, and PAHs from 9 laboratories. Two laboratories that received PUF samples did not report any results.

### 2.3 Phase 3 – different samplers, different PUFs, different laboratories

The objective of Phase 3 was to identify the full variability in SVOC measurements due to the combined effect of different sampler designs and laboratory analyses. This reflects the “realistic” variability that would occur between different studies/monitoring networks. In this Phase, 14 laboratories sent a PAS housing and PUF disk to NILU, and each laboratory’s own PUF-PAS configuration (considering housing, PUF disk and installation parameters) was deployed at the Norwegian field site (deployment height 2 m), concurrent with Phase 2 from September 17, 2015 to December 3, 2015. After 77 days the PUF disk and a corresponding field blank were collected and shipped with the Phase 2 samples. As with Phase 2, participating laboratories were asked to analyze the PUFs for seven PCB congeners, 10 OCPs, 10 PBDE congeners and 16 PAHs (Table S5) and report results to an Excel template. Laboratories used the same analytical methods as for Phase 2 (Table S8) and reported identical sets of compounds, resulting in records for PCBs from 11 laboratories, OCPs from 11 laboratories, PBDEs from 10 laboratories, and PAHs from 9 laboratories. Two laboratories that received PUF samples did not report any results.

### 2.4 Quality assurance/quality control

Each participating laboratory reported their internal standards, instrumental detection limits, and method detection limits for Phases 2 and 3.

All PUFs were sequentially pre-cleaned by Soxhlet at NILU laboratories with 24 h toluene, 8 h acetone, 8 h hexane, and then dried under vacuum. Field blanks were included in all three phases. Each PUF disk sample sent to participating laboratories was paired with a field blank of the same PUF disk type. PUF disks were only numbered and were not separately identified as field blank or sample. All field blanks were pre-cleaned at the same laboratory (NILU), using the same method. Thus, any variability in levels in the field blanks should be due to contamination during transport or laboratory procedures.

Data received from the Excel template spreadsheets were compiled separately for each compound group. Each sampler was assigned a number code for Phase 1 data (1–16) and each laboratory was assigned a letter code for Phases 2 and 3 data (A—M) to anonymize all results. Inconsistencies or missing values in reported data were addressed individually with participating laboratories. Data handling and statistical evaluation was done through MS Excel and R software.

## 3 Results and discussion

Twelve different sampler designs were received, differing in dome shape, internal volume, overhang, and gap diameter. Not all domes were hemispherical (some had a straight-sided conical shape), thus individual dome volumes were measured based on the mass of water that could fill each dome. Dome gap dimensions and overhangs were measured for the assembled sampler design, and the surface areas of the gap between upper and lower dome, i.e., the main space for air diffusion into the PAS housing, were calculated assuming circular geometry. All sampler housings also allowed additional diffusion through holes in the bottom of the lower dome, although the number of holes varied from 4 to 8 depending on the sampler. Samplers and associated indicators of sampler geometry are shown in Fig. 1. Internal volumes (V) ranged from 3340 to 6750 cm^3^, and surface areas of the gap between upper and lower dome (G) from 75 to 171 cm^2^ (Fig. 1). Differences in the PUF disks were smaller, with diameters of 13.2–14.1 cm, thicknesses of 1.1–1.5 cm, and densities of 0.020 to 0.031 g/cm^3^. Full dimensions of samplers and PUF disks are given in Table S2.

### 3.1 Phase 1 – different samplers, same PUFs, same laboratory

The objective of Phase 1 was to isolate and identify the influence of different sampler designs on sampler uptake by comparing different housings fitted with identical PUF disks, deployed simultaneously at the same site, and analyzed in a single laboratory. The differences in the masses of SVOCs sampled by each PAS should therefore give insight into the variability in uptake introduced only by sampler geometry (dome sizes, gap and overhang) and deployment (e.g., fixed vs. free swinging).

Compounds that were below detection limits in 50 % or more of the PAS were excluded from further interpretation. This resulted in the exclusion of eight of 29 PAHs (naphthalene, biphenyl, acenaphthylene, cyclopenta(cd)pyrene, perylene, dibenzo(ah)anthracene, dibenzo(ac)anthracene, and anthanthrene), five of 12 OCPs (β- and δ-HCH, and o,p’-DDD, p,p’-DDD and o,p’-DDE), two of seven PCBs (PCB-138, PCB-180), and six of ten PBDEs (BDE-66, BDE-85, BDE-99, BDE-100, BDE-153, BDE-154). Any results <MDL for the remaining compounds were substituted with 0.5*MDL for statistical analysis. For PAHs and PCBs, this resulted in substitution of 3% of records, and no substitutions for OCPs; for PBDEs the substitution was required for 37 % of records, thus there is higher uncertainty in the statistical analysis of the PBDEs than for the other compound groups.

**Fig. 2:**
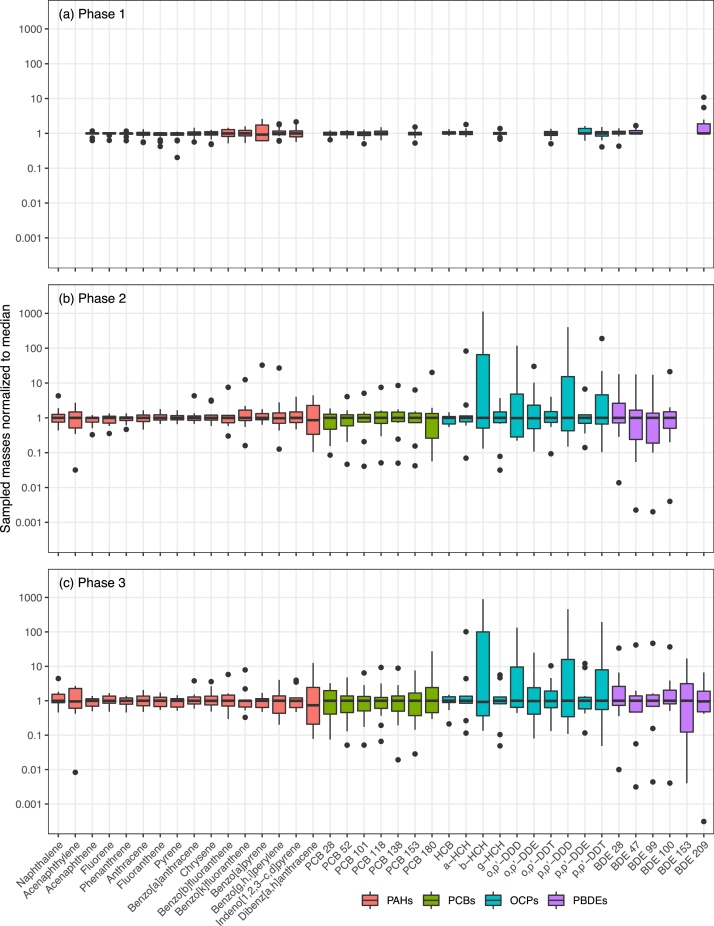
Variability in analytes detected by PUF-PAS due to differences in (a) sampler geometry and installation (Phase 1, n = 16), (b) analytical methods between laboratories (Phase 2, n = 13), and (c) combined sampler and analytical differences (Phase 3, n = 13). Boxes represent the 25^th^ to 75^th^ percentiles, with the median (50^th^ percentile) as a horizontal black line. Individual SVOC levels were normalized to the median. Whiskers represent ±1.5 times the interquartile range (IQR) with individual points indicating outliers.

We evaluated the variability in the individual samplers by normalizing masses measured per compound to the median masses for all samplers (Fig. 2a). Compounds were mostly within a relatively narrow range, spanning 519–939 ng/PUF for Σ_21_PAHs (Table S9), 4.86–12.3 ng/PUF for Σ_6_PCBs (Table S10), 0.80–2.21 for Σ_6_DDXs (Table S11), 2.54–5.50 ng/PUF for Σ_4_HCHs (Table S11), and 59.5–109 pg/PUF for Σ_9_PBDEs (excluding BDE-209; range for BDE-209 was < MDL–1300 pg/PUF) (Table S12). Individual compound masses were within one order of magnitude of the median for all of the sampler designs, with the exception of one record of BDE-209 (Fig. 2a). In general, the variability increased with increasing molecular weight of compounds, e.g., high molecular weight PAHs and PBDEs had the highest variability.

Five of the samplers (Samplers 1–5) were identical configurations of the commercially available Tisch sampler design (TE-200, Tisch Environmental, Cleves, OH). We considered these samplers as replicates and used them to assess the typical range of variability between identical co-deployed samplers (i.e., due to environmental conditions and laboratory uncertainty rather than differences in sampler configuration). These Tisch replicates were normally distributed (Shapiro-Wilks test, α = 0.05, except for p,p’-DDE, and BDE-47 and 209), so means and standard deviations were used to evaluate their distribution. The relative standard deviation (RSD) of the five replicates ranged from 1.98 % for acenaphthene to 55.6 % for BDE-183 (Table S13). The highest RSDs were observed for the higher molecular weight compounds, i.e. 5- and 7-ring PAHs and BDEs 183 and 209 (average 22.7 % vs. 8.9 % for all other compounds), which likely reflects two possible effects on higher molecular weight compounds: (1) higher analytical uncertainty and (2) variable sampler uptake of particulates. Lower ambient levels of higher molecular weight compounds may lead to greater measurement uncertainties as MDLs are approached. The larger variability in the uptake of particle-bound compounds to PUF-PAS has been extensively discussed in other publications (e.g., Holt et al., 2017; Markovic et al., 2015). However, we note that the Tisch sampler is reported as having high particle infiltration in Markovic et al. (2015), suggesting that even when particle infiltration is high, there remains higher variability/uncertainty for particle uptake than gaseous compounds.

The RSD determined from the Tisch samplers was assumed to represent the typical uncertainty in a sampler due to environmental variability and laboratory uncertainty and was therefore used to flag cases when the variability between PAS was beyond the range of typical differences between identical samplers. Upper and lower boundaries were calculated per compound as the median of all sampler masses ±3 times the percent uncertainty (Tables S9-S12). We identify the specific cases and compounds where variability exceeded these thresholds.

**Fig. 3:**
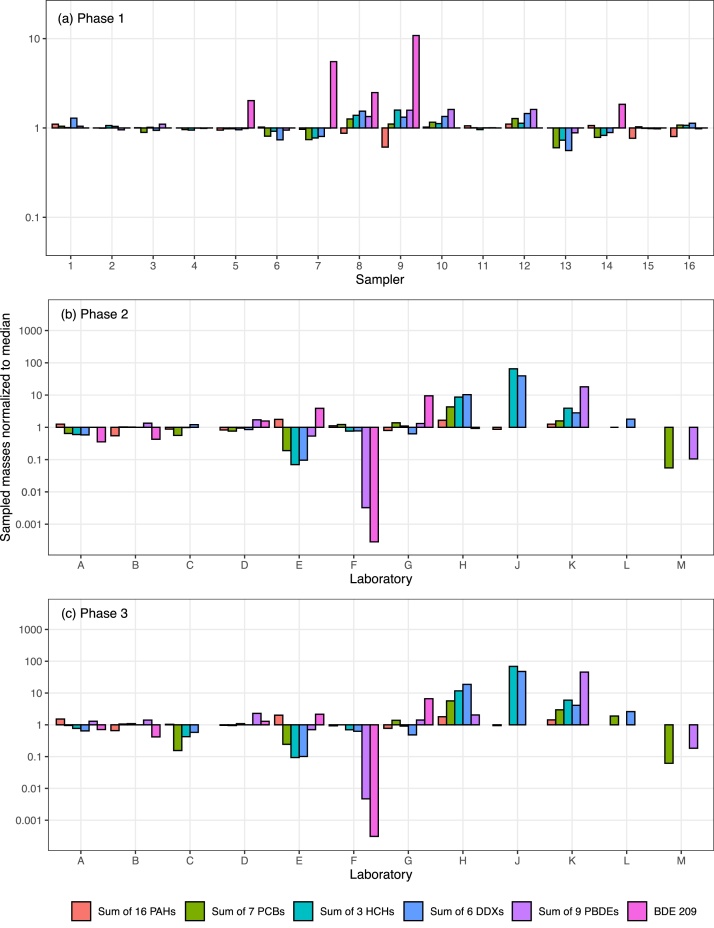
Reported SVOC masses normalized to median for (a) Phase 1, (b) Phase 2, and (c) Phase 3. Phase 1 shows variability between different PUF-PAS sampler designs (1–16) analyzed at a single laboratory; Phase 2 shows variability between co-deployed, identical PUF-PAS samplers analyzed in 13 different laboratories (A–M); and Phase 3 shows differences in co-deployed different samplers analyzed in different laboratories (Labs A-M). Samplers 1–5 in Phase 1 are identical Tisch TE-200 samplers. Note the smaller y-axis scale in Phase 1 compared to Phases 2 and 3.

No single sampler design had all results within the acceptable range (median±3xRSD of Tisch samplers), however most samplers had only a few compounds outside of this range (Tables S9-S12), with the Tisch samplers (Samplers 1–5) demonstrating the least variability across all compound groups (Fig. 3a). A few samplers had more substantial deviations from the median values: Sampler 6 measured 24 % lower total SVOC masses than the median, with OCPs particularly low; Sampler 13 recorded 38 % lower total SVOC masses, with OCPs and PCBs particularly low (Fig. 3a); Sampler 8 recorded 11 % higher total SVOC masses (Fig. 3a). BDE-209 was substantially more variable than other compounds, with most cases < MDL and five samplers with notably high values.

We also examined the correlation between the measured masses and the sampler geometry parameters. The strongest correlation was with the overhang distance (i.e. the overlap between the top and bottom domes), with a significant negative relationship between overhang distance and mass of SVOCs collected by the PUF (Spearman ρ of -0.796, p < 0.01 for correlation between overhang distance and total SVOCs), e.g., larger overhang is correlated with lower mass. This suggests reduced airflow to the inside of the sampler housing due to overhang, and consequent lower uptake of compounds, especially particle-bound compounds (Markovic et al., 2015). Despite large ranges in the other sampler geometries (e.g., sampler volumes ranging from 3340 to 6750 cm^3^), no other sampler geometry parameters had significant correlations with the mass of SVOCs collected.

While individual differences were generally small, small systematic errors across many individual compounds can lead to significant differences in compound group totals, e.g., indicators such as ΣEPA-16 PAHs and Σ_7_PCBs, which are frequently applied in policy and effectiveness evaluation. For example, ΣEPA-16 PAHs is 482 ng/PUF in the lowest reporting sampler (Sampler 13) vs. 873 ng/PUF in the highest (Sampler 10), and for Σ_7_PCB the mass collected was 2440 pg/PUF vs. 5120 pg/PUF in Samplers 13 vs. 8. While these are the extremes of the set, it does suggest that uncertainties introduced by differences in sampler configuration can account for up to 50 % variation in reported sample masses. Moreover, it is known that at sites with meteorological extremes (e.g., coastal sites with very high wind speeds, polar sites) the protective effect of the sampler housing on PAS uptake rates is variable, and the effects of differences between sampler configurations could be exacerbated, leading to variation beyond what was seen in this test case.

### 3.2 Phase 2 – same samplers, same PUFs, different laboratories

Results for Phase 2 indicate the extent of variability between SVOC measurements introduced by differences in transport, laboratory handling and analysis of PUF disks. Major differences were identified in two aspects: field blank contamination and sample masses.

Field blank masses varied over 2 orders of magnitude, spanning 187–8260 pg/PUF for Σ_7_PCBs, <3–640 pg/PUF for HCB, 50–35600 pg/PUF for Σ_3_HCHs, <50–98400 pg/PUF for Σ_6_DDXs, 17.9–1670 ng/PUF for Σ_16_PAHs, and <25–3250 pg/PUF for Σ_10_PBDEs (Table S14). The 2–3 order of magnitude range in field blanks suggests either errors in analysis and/or large variations in contamination during transport and processing, with the implication being that choices regarding blank treatment can have a large impact on reported values. We also note that in three cases, masses of PBDEs and DDXs reported in field blanks exceeded those reported in the samples (Fig. S2). PBDEs and other flame retardants are often identified as a particular challenge for analysis in PUF samples due to their prevalence in equipment and electronics, and low ambient levels at many locations, including the NILU site in this study.

Despite the significant contribution of blanks to total samples for a few laboratories (laboratories A, J, K; Fig. S1), we did not further adjust samples for blanks as we had only one field blank per laboratory, and in practice many laboratories use blanks only as a quality control in long-term monitoring, rather than for data adjustment.

Phase 2 sample results clearly indicate that the variability due to laboratory analysis is much higher than that introduced by sampler geometry identified in Phase 1. The Σ_16_PAH reported spanned 1830–5870 ng/PUF, a substantially larger span than that observed in Phase 1 (Table S15). Variations were even higher for POPs, with reported values spanning 3 or more orders of magnitude: Σ_3_HCHs spanning 283–263000 pg/PUF, Σ_6_DDX from 171 to 70100 pg/PUF (Table S16), Σ_7_PCBs from 378 to 29300 pg/PUF (Table S17) and Σ_10_PBDEs from 0.35 to 1950 pg/PUF (Table S18).

We explored whether the choice not to adjust sample masses for field blank contamination led to such large ranges, but the effect was limited. For example, when blanks were subtracted from measured values Σ_7_PCBs ranged 378–23000 pg/PUF, Σ_3_HCHs ranged 243–227000 pg/PUF, Σ_6_DDX ranged 140–14200 pg/PUF, and the range for PBDEs did not change, suggesting that the large variability in their reported masses is independent of differences in blank contamination.

As with Phase 1, we assessed the variability by normalizing sampled SVOC masses to the median of the whole set of reported masses (Fig. 2b). It is clear from the range of the normalized data, with some compounds covering a range of ∼0.1–1000 around the median, that much greater differences in reported masses are introduced by laboratory analysis than by differences in sampler design (where the range of normalized masses was much less than 0.1–10 times the median, Fig. 2a). We note that some of the compounds with the largest ranges in Phase 2 (e.g., PCB 180, dibenzo[a,h]anthracene, β-HCH) were below detection in Phase 1 in all samples, which inherently suggests that the variability for these compounds is smaller than the LOD. Of the compounds detected in both phases, all compounds except chrysene had at least a 2x higher span in reported values in Phase 2 compared with Phase 1, and the span of values was more than 50x higher for BDE-28, BDE-47 and p,p’-DDT.

This substantial increase in variability in Phase 2 compared with Phase 1 indicates that, due to the uncertainty introduced by analysis in different laboratories, close to 50 % of laboratories report SVOC masses with order of magnitude differences from a consensus values, i.e., median of all laboratories (Fig. 3b). We compared the deviations from consensus values with the analytical methods used across the laboratories (Table S8) but did not find any relationship with the basic parameters of extraction method, solvent, clean-up or instrumental analysis and the laboratory performance. However, it is clear from the difference between Phases 1 and 2 that the sample processing and analysis are an important contributor to the differences in the reported values between laboratories. We are unable to identify specific method influences on performance due to the large differences in methods used across the 12 laboratories (e.g., eight different extraction methods), which prevents us from making generalizations.

The differences introduced by analytical methods are higher than what has been typically identified by global interlaboratory evaluations on POPs, e.g., the UNEP-supported Bi-ennial Global Interlaboratory Assessments on POPs (Nilsson et al., 2014; van Bavel et al., 2012; van der Veen et al., 2017; Van Leeuwen et al., 2013). However, the Bi-ennial Global Interlaboratory Assessments on POPs included injection-ready test mixtures and environmental matrices high in organic matter or lipid content, or else spiked air samples (Fiedler et al., 2020, 2017; Nilsson et al., 2014), with relatively high concentrations. These concentrations were higher than what are found in typical rural/remote air samples, and many interlaboratory evaluations have reported lower precision and accuracy at lower concentrations (Melymuk et al., 2018, 2015; Su and Hung, 2010). The low concentrations in our samples collected from semi-rural Norway likely contributed to the poorer performance in our study. Further, our study captured the full scope of variability in sample processing, as laboratories received the PUF material, and were required to extract and purify the samples. This contrasts with the Bi-ennial Global Interlaboratory Assessments on POPs, where laboratories analyzed air extracts not requiring further clean-up (van der Veen et al., 2017); the variability introduced from extraction and clean-up of complex matrices is a large contributor to the differences between laboratories (Abalos et al., 2013; Melymuk et al., 2018; Su and Hung, 2010; Van Leeuwen et al., 2013).

If these differences reflect a recurring deviation by certain laboratories, this suggests that international comparisons of SVOC data could be highly biased when analyses are performed at different laboratories. This does not impact internal reporting within individual laboratories, therefore comparability within individual laboratory research studies and monitoring programs, (e.g., to assess regional spatial differences or temporal changes), should still be valid. Yet when comparisons involve merging data from multiple studies/monitoring networks, (e.g., for model comparisons), there may be significant biases introduced in the interpretation on a global level. This may also apply to data reported from active samplers, which typically follow similar methods of laboratory analysis.

### 3.3 Phase 3 – different samplers, different PUFs, different laboratories

Phase 3 gives an indication of overall variability, encompassing differences between sampler configurations identified in Phase 1 and between laboratory analysis identified in Phase 2. As with Phase 2, large differences were observed in both field blanks and reported sample masses. Field blanks had a similar distribution and range to those of Phase 2 (Table S19) and in two cases the blank mass exceeded that of the sample (Fig. S3). As in Phase 2, no blank adjustment was applied to the reported sample masses.

Phase 3 showed variability in sample masses comparable to that of Phase 2. The Σ_16_PAH ranged 1640–5010 ng/PUF (Table S20), a similar range to that observed in Phase 2. As in Phase 2, variations were higher for POPs, with ranges spanning >3 orders of magnitude: from 247 to 182000 pg/PUF for ΣHCH, 123–58200 pg/PUF for ΣDDX (Table S21), 283–25900 pg/PUF for Σ_7_PCBs (Table S22), and 0.44–3110 pg/PUF for ΣPBDEs (Table S23). As with Phases 1 and 2, we assessed the variability by normalizing masses to the median of the whole set of reported masses (Fig. 2c). The range of the normalized data, covering ∼0.001–1000 around the median, was similar to Phase 2 (Fig. 2b), and much greater than Phase 1 (Fig. 2a).

Phase 3 combined three factors affecting variability (1) sampler design, (2) sample transport and laboratory analysis, and (3) differences in PUF disk parameters (size, density; Table S2). While we did not have a separate study phase to isolate the influence of differences in PUF disks, the similarity of results from Phases 2 and 3 suggest that this is minimal, and likely comparable to or less than the variability introduced by sampler design. This is supported by previous work identifying 40–60 % differences in particle uptake and distribution in high density vs. low density PUF disks (Chaemfa et al., 2009), which was a larger range in density than that seen within our study.

As in Phase 2, a large fraction of laboratories reported masses that differed by more than one order of magnitude from the median (Fig. 3c), suggesting that, when PUF-PAS data are reported by different laboratories using different samplers, ∼50 % of laboratories are reporting values outside the boundaries of acceptable uncertainty, which can create a major challenge in the global comparability of SVOC data from PUF-PAS monitoring.

The similarity in the ranges and collected masses between Phases 2 and 3 suggests the major influence on differences in comparability between laboratories is sample transport, processing and analytical variation, and this source of variability overwhelms any smaller differences due to sampler design identified in Phase 1.

## 4 Conclusions and implications

Phase 1 of this international intercomparison revealed that variations in the double-dome PAS housings used by different research groups contributes relatively little to uncertainties in sampled masses of PCBs, PAHs, PBDEs, and OCPs for a 3-month deployment, with differences in reported masses due to PUF-PAS sampler configurations not exceeding 50 %. Any difference in uptake between samplers appears related to differences in airflow into the sampler housing due to the amount of overhang of the upper dome over the lower.

Phase 2 of the study, which assessed uncertainty associated with laboratory performance (i.e., each laboratory analyzing the same type of sample), showed a substantial increase in uncertainty. Reported masses varied by an order of magnitude or greater, with ∼10× differences between individual masses and medians for many compounds, as well as a few more extreme outliers (Fig. 2). Phase 3 of the study confirmed the results of Phase 2; similar ranges of reported values and large deviations from medians for some individual laboratories/compounds show that the major influence on comparability between laboratories is sample processing and analytical variation, and this source of variability overwhelms any smaller differences due to sampler design and differences in PUF disk size and density. Considering only the three laboratories that currently report PUF-PAS data to the Stockholm Convention GMP, the variability is lower, but there still exist order-of-magnitude differences in reported masses of Σ_7_PCBs, HCB and Σ_6_DDX.

The high level of uncertainty observed in Phases 2 and 3 of this study indicates that current global air monitoring data are not directly comparable between different laboratories/monitoring programs for most of the SVOCs included in this study. However, this does not mean that the data are not internally consistent (i.e., within a program using a single laboratory) for deriving valid spatial and temporal trends. Yet on a global scale, it is clear that merging data from multiple laboratories must be done with caution. With current levels of uncertainty, it is not feasible to compare results between laboratories/monitoring programs without prior assurance of comparability in reported data. It is also clear that the uncertainty is not limited to passive sampling but also pertains to active air sampling results if similar extraction, clean-up and analytical procedures are followed. This uncertainty may be due to several factors including instrumental methods, sample processing procedures, potential laboratory contamination due to solvents and other sources, and differences in analytical standards (not assessed in the current study). Additional uncertainties will also arise if concentrations are adjusted to volumetric units (e.g., pg/m^3^), since slightly different conventions may be used among laboratories for estimating effective air sample volumes. These uncertainties can be resolved through ongoing participation in intercalibration exercises and adoption of best practices. The high variability between laboratories means that a crucial part of any efforts to integrate and evaluate global spatial patterns of POPs in air must require and implement intercalibration to assess and account for uncertainties, repeated at regular intervals for both active and passive air sampling. Some examples of such international repeated intercalibration exercises already exist, most notably the AMAP/EMEP intercomparison exercises (Schlabach et al., 2012; Tkatcheva et al., 2013). Establishing such actions as a part of research infrastructure is even more necessary given the efforts to mobilize data from additional monitoring networks to contribute to global data repositories.

Effectiveness evaluation of international SVOC actions (Stockholm Convention, CLRTAP) relies on the provision of high-quality air monitoring data. PUF-PAS are a valuable tool to provide this information, particularly given that the slight differences in PUF-PAS sampler designs do not greatly affect data comparability. Yet without establishment of frameworks to ensure analytical comparability, our understanding of global spatial POP distributions will be biased by the regional differences in analytical performance.

## CRediT authorship contribution statement

**Lisa Melymuk –** Conceptualization, Methodology, Writing, Formal Analysis; **Pernilla Bohlin Nizzetto–** Conceptualization, Methodology, Resources, Review & Editing; **Tom Harner** – Conceptualization, Resources, Review & Editing, Supervision; **Kevin B. White** – Formal Analysis, Visualization, Review & Editing; **Xianyu Wang** – Investigation, Resources, Review & Editing; **Maria Yumiko Tominaga** – Investigation, Resources, Review & Editing; **Jun He** – Investigation, Resources, Review & Editing; **Jun Li –** Investigation, Resources, Review & Editing; **Jianmin Ma** – Investigation, Resources, Review & Editing; **Wan-Li Ma** – Investigation, Resources, Review & Editing; **Beatriz H. Aristizábal** – Investigation, Resources, Review & Editing; **Annekatrin Dreyer** – Investigation, Resources, Review & Editing; **Begoña Jiménez** – Investigation, Resources, Review & Editing; **Juan Muñoz-Arnanz** – Investigation, Resources, Review & Editing; **Mustafa Odabasi** – Investigation, Resources, Review & Editing; **Yetkin Dumanoglu** – Investigation, Resources, Review & Editing; **Baris Yaman** – Investigation, Resources, Review & Editing; **Carola Graf** – Investigation, Resources, Review & Editing; **Andrew Sweetman –** Investigation, Resources, Review & Editing; **Jana Klánová** – Resources, Supervision, Review & Editing.

## Declaration of Competing Interest

The authors report no declarations of interest.
