# Supplementary material for: Global intercomparison of polyurethane foam passive air samplers evaluating sources of variability in SVOC measurements
Source: Environ Sci Policy. 2021 Nov;125:1–9. doi: 10.1016/j.envsci.2021.08.003 (PMC8525512; doi:10.1016/j.envsci.2021.08.003)
Supplement: Supplementary file 1 [file mmc1.pdf]

Supporting Information:

Global intercomparison of polyurethane foam passive air samplers evaluating sources of variability in SVOC measurements

Lisa Melymuk[1], Pernilla Bohlin Nizzetto[2], Tom Harner[3], Kevin B White[1], Xianyu Wang[4], Maria Yumiko Tominaga[5], Jun He[6], Jun Li[7], Jianmin Ma[8], Wan-Li Ma[9], Beatriz H. Aristizábal[10], Annekatrin Dryer[11], Begoña Jiménez[12], Juan Muñoz-Arnanz[12], Mustafa Odabasi[13], Yetkin Dumanoglu[13], Baris Yaman[13], Carola Graf[14], Andrew Sweetman[14], Jana Klánová[1]

1 - RECETOX, Masaryk University, Brno, Czech Rep.; 2 - NILU - Norwegian Institute for Air Research, Kjeller, Norway; 3 - Air Quality Processes Research Section, Environment and Climate Change Canada, Toronto, Canada; 4 - Queensland Alliance for Environmental Health Sciences (QAEHS), The University of Queensland, Australia; 5 - CETESB - São Paulo State Environmental Company, São Paulo, Brazil; 6 - Department of Chemical and Environmental Engineering, University of Nottingham Ningbo China, Ningbo, China; 7 - State Key Laboratory of Organic Geochemistry, Guangzhou Institute of Geochemistry, Chinese Academy of Sciences, Guangzhou, China; 8 - College of Urban and Environmental Sciences, Peking University, Beijing, China; 9 - International Joint Research Center for Persistent Toxic Substances (IJRC-PTS), Harbin Institute of Technology, Harbin, China; 10 - Hydraulic Engineering and Environmental Research Group (GTAIHA), Universidad Nacional de Colombia, Manizales, Colombia; 11 - ANECO Institut für Umweltschutz, Hamburg, Germany; 12 - Department of Instrumental Analysis and Environmental Chemistry, IQOG-CSIC, Madrid, Spain; 13 - Department of Environmental Engineering, Dokuz Eylul University, Buca-Izmir, Turkey; 14 - Lancaster Environment Centre, Lancaster University, UK

List of Tables

Table S1: Participating research groups S3
Table S2: Sampler dimensions and properties S4
Table S3: Meteorological conditions during Phase 1 deployment in Kjeller, Norway S5
Table S4: Meteorological parameters during Phase 2 and 3 deployments, Kjeller, Norway S6
Table S5: Target compounds according to study phases S7
Table S6: Percentage recoveries for Phase 1 samples (n=16) S9
Table S7 - Field blank levels and method detection limits for Phase 1. .... S9
Table S8 – Analytical methods used by participating laboratories S10
Table S9 - Phase 1 PAH masses (ng/sampler) flagged if outside of typical sampler variability S11
Table S10 - Phase 1 PCB masses (pg/sampler) flagged if outside of typical sampler variability S12
Table S11 - Phase 1 OCP masses (pg/sampler) flagged if outside of typical sampler variability S12
Table S12 - Phase 1 PBDE masses (pg/sampler) flagged if outside of typical sampler variability S13
Table S13 - Assessment of variability in 5 identical Tisch samplers in Phase 1 .... S14
Table S14: Phase 2 field blanks S15
Table S15 - Phase 2 PAH masses S17
Table S16 - Phase 2 OCP masses S18
Table S17 - Phase 2 PCB masses S18
Table S18 - Phase 2 PBDE masses. S19
Table S19: Phase 3 field blanks by Lab ID. S19
Table S20 - Phase 3 PAH masses (ng/sampler) S21
Table S21 - Phase 3 OCP masses (pg/sampler) S22
Table S22 - Phase 3 PCB masses (pg/sampler) S22
Table S23 - Phase 3 PBDE masses (pg/sampler) S23

List of Figures

Figure S1: PUF-PAS sites of networks reporting to Stockholm Convention GMP as of 2018. .... S3
Figure S2: Deployment locations of PUF-PAS, indicated by the orange line. S7
Figure S3: Percentage contribution of field blanks to total sample mass, Phase 2. .... S17
Figure S4: Percentage contribution of field blanks to total sample mass, Phase 3. .... S21

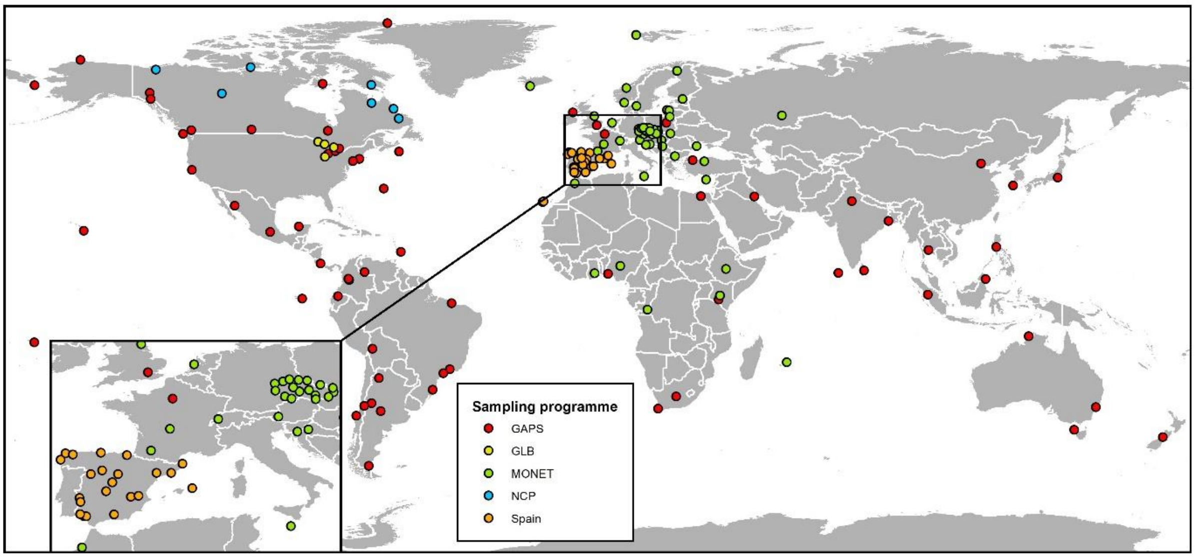

Figure S1: PUF-PAS sites of networks reporting to Stockholm Convention GMP as of 2018. GAPS is the Global Atmospheric Passive Sampling network, GLB is the Great Lakes Basin sampling network, NCP is the Northern Contaminants Program, all the responsibility of Environment and Climate Change Canada. MONET is the Monitoring NETwork of Masaryk University. Spain refers to the Spanish passive sampling network, of CSIC.

Table S1: Participating research groups

**Country**	**Research Centre**
Australia	Queensland Alliance for Environmental Health Sciences, University of Queensland
Brazil	Environmental Agency of Sao Paulo State (CETESB)
Canada	Environment and Climate Change Canada/Global Atmospheric Passive Sampling network
China	Lanzhou University
China	Guangzhou Institute of Geochemistry
China	Harbin Institute of Technology
China	The University of Nottingham, Ningbo
Colombia	Universidad Nacional de Colombia, Manizales, Colombia
Czech Republic	RECETOX, Masaryk University/MONET Networks
Germany	Eurofins GfA GmbH (2 samplers)
Mexico	Center for Atmospheric Sciences, National Autonomous University of Mexico
Norway	Norwegian Institute for Air Research (NILU)
Spain	Institute of Organic Chemistry (IQOG), Spanish National Research Council (CSIC)
Turkey	Dokuz Eylul University
UK	Lancaster Environment Centre, Lancaster University

Table S2: Sampler dimensions and properties

	Upper Dome			Lower Dome				Whole sampler							PUF					
Sampler ID	**Diameter (cm)**	**Depth (cm)**	**Volume (cm^3)**	**Diameter (cm)**	**Depth (cm)**	**Volume (cm^3)**	**Holes in lower**	**Upper/ lower dome ratio**	**Total volume (cm^3)**	**Overhang (cm)**	**Horizontal gap (cm)**	**Assemble d volume (cm^3)**	**Gap surface area (cm^2)**	**Internal vol/gap surface**	**Diameter- long (cm)**	**Diameter- short (cm)**	**Thickness (cm)**	**Mass (g)**	**Volume (cm^3)**	**Density (g/cm^3)**
1	23.5	8.0	2300	18.5	7.0	1240	7	1.85	3540	0.8	2.0	3338	135.1	24.7	13.7	13.3	1.2	4.8	171.7	0.028
2	23.5	8.0	2300	18.5	7.0	1240	7	1.85	3540	0.8	2.0	3338	135.1	24.7	13.7	13.3	1.2	4.8	171.7	0.028
3	23.5	8.0	2300	18.5	7.0	1240	7	1.85	3540	0.8	2.0	3338	135.1	24.7	14.3	13.0	1.2	4.5	175.2	0.026
4	23.5	8.0	2300	18.5	7.0	1240	7	1.85	3540	0.8	2.0	3338	135.1	24.7	14.0	14.0	1.2	4.7	185.8	0.025
5	23.5	8.0	2300	18.5	7.0	1240	7	1.85	3540	0.8	2.0	3338	135.1	24.7	14.0	14.0	1.2	4.7	185.8	0.025
6	25.7	8.4	3150	19.7	7.0	1400	4	2.25	4550	1.0	2.1	4245	155.7	27.3	13.9	13.4	1.3	4.7	190.2	0.025
7	21.0	11.3	3100	16.5	9.4	1550	8	2.00	4650	0.5	1.2	4543	74.6	60.9	14.0	14.0	1.2	4.7	185.8	0.025
8	24.5	10.0	3340	18.9	7.8	1800	4	1.86	5140	0.5	1.0	5000	73.8	67.7	14.0	14.0	1.2	4.7	185.8	0.025
9	24.0	10.0	3800	17.5	8.0	1660	6	2.29	5460	1.2	1.8	5171	125.5	41.2	13.4	14.1	1.3	3.9	192.9	0.020
10	24.5	10.0	3420	18.9	7.8	1860	4	1.84	5280	0.0	1.9	5280	134.9	39.1	13.6	13.6	1.5	6.0	217.9	0.028
11	26.1	10.5	3920	19.4	6.8	1640	4	2.39	5560	0.4	2.2	5442	165.2	32.9	14.0	14.0	1.2	4.7	185.8	0.025
12	24.5	10.0	3400	18.9	7.8	1860	4	1.83	5260	-0.9	1.8	5512	128.4	42.9	14.0	14.0	1.2	4.7	185.8	0.025
13	27.8	10.6	4920	21.3	5.9	1600	4	3.08	6520	2.3	2.1	5700	169.6	33.6	13.2	13.9	1.1	4.9	158.5	0.031
14	23.6	11.0	4150	19.8	9.4	2560	8	1.62	6710	0.8	1.1	6479	77.8	83.3	14.0	14.0	1.2	4.7	185.8	0.025
15	28.2	10.7	4500	22.1	8.1	2180	4	2.06	6680	0.0	1.8	6680	149.3	44.7	14.0	14.0	1.2	4.7	185.8	0.025
16	28.0	10.6	4600	22.8	7.4	2150	4	2.14	6750	0.0	2.1	6750	170.9	39.5	14.1	14.1	1.4	5.9	218.6	0.027

Table S3: Meteorological conditions during Phase 1 deployment in Kjeller, Norway

Date	Daily average temp. (˚C)	Daily min. temp. (˚C)	Daily max. temp. (˚C)	Daily avg. wind speed (m/s)
2016-04-01	3.1	-4.6	10.0	2.3
2016-04-02	4.7	2.2	6.8	2.8
2016-04-03	5.1	3.7	6.3	1.7
2016-04-04	6.2	4.3	8.4	2.1
2016-04-05	6.3	4.9	8.6	1.9
2016-04-06	6.3	4.3	10.1	1.9
2016-04-07	5.4	3.5	8.4	1.5
2016-04-08	4.3	3.0	5.9	2.0
2016-04-09	4.7	2.6	10.4	1.9
2016-04-10	4.9	-2.5	12.7	1.6
2016-04-11	5.3	-0.8	12.7	1.3
2016-04-12	6.0	-2.2	14.0	1.5
2016-04-13	6.4	-2.2	13.0	2.6
2016-04-14	6.4	3.5	10.5	3.1
2016-04-15	6.9	5.2	10.0	3.0
2016-04-16	6.7	3.9	11.0	2.2
2016-04-17	4.6	2.2	8.1	4.1
2016-04-18	6.7	1.9	12.2	3.4
2016-04-19	7.4	0.0	13.9	4.4
2016-04-20	7.1	-0.8	13.4	2.2
2016-04-21	9.1	3.4	15.0	4.0
2016-04-22	5.3	1.3	10.1	4.1
2016-04-23	2.7	-4.1	8.5	2.2
2016-04-24	2.8	-1.9	8.8	2.6
2016-04-25	2.7	-2.5	8.6	2.4
2016-04-26	3.3	-4.3	9.2	2.9
2016-04-27	4.4	-4.4	11.4	1.7
2016-04-28	3.9	-1.6	9.3	3.7
2016-04-29	5.2	0.4	9.7	4.0
2016-04-30	2.5	0.3	7.5	1.6
2016-05-01	5.9	1.8	10.4	2.2
2016-05-02	7.9	2.2	11.2	3.1
2016-05-03	8.5	6.9	12.1	3.1
2016-05-04	8.8	0.6	16.1	2.3
2016-05-05	9.1	1.1	15.9	2.9
2016-05-06	10.5	1.5	17.6	2.3
2016-05-07	12.7	2.5	21.6	2.0
2016-05-08	15.3	3.7	23.4	2.2
2016-05-09	16.4	3.5	25.3	1.6
2016-05-10	16.4	5.1	24.5	2.4
2016-05-11	12.8	6.5	20.9	3.0
2016-05-12	13.5	3.4	20.2	3.2
2016-05-13	8.6	5.1	16.3	5.2
2016-05-14	8.7	4.7	13.3	5.1
2016-05-15	9.8	4.9	14.5	5.6
2016-05-16	10.9	5.3	16.5	4.2
2016-05-17	10.9	1.1	17.4	2.4
2016-05-18	11.7	7.1	16.9	2.6
2016-05-19	12.3	8.4	17.9	2.3
2016-05-20	11.3	9.5	14.2	2.6
2016-05-21	11.7	9.0	16.8	2.7
2016-05-22	13.5	10.5	15.6	2.0
2016-05-23	13.8	13.7	15.4	2.1
2016-05-24	9.9	8.5	13.8	3.9
2016-05-25	12.8	8.2	17.9	5.1
2016-05-26	13.0	5.7	18.8	3.4
2016-05-27	14.7	10.3	20.4	3.4
2016-05-28	13.2	9.7	17.3	2.4
2016-05-29	13.2	12.3	16.1	3.2
2016-05-30	15.2	12.4	20.7	3.6
2016-05-31	19.1	10.5	26.2	2.3
2016-06-01	20.9	12.4	27.5	1.7
2016-06-02	21.0	13.8	27.9	1.8
2016-06-03	21.0	13.2	28.5	2.1

Table S4: Meteorological parameters during Phase 2 and 3 deployments, Kjeller, Norway

Date	Daily average temp. (˚C)	Daily min. temp. (˚C)	Daily max. temp. (˚C)	Daily avg. wind speed (m/s)
2015-09-11	11.8	6.0	19.0	1.8
2015-09-12	12.5	7.6	15.0	2.9
2015-09-13	14.0	11.8	16.8	3.5
2015-09-14	14.3	12.7	16.0	4.9
2015-09-15	12.3	11.2	15.4	3.4
2015-09-16	10.6	6.1	13.1	1.6
2015-09-17	13.6	11.4	17.0	2.7
2015-09-18	14.4	12.8	19.1	2.1
2015-09-19	11.7	6.3	18.8	1.2
2015-09-20	9.2	2.7	14.3	1.0
2015-09-21	10.4	7.1	15.9	1.1
2015-09-22	9.6	5.3	11.8	1.3
2015-09-23	11.4	7.9	14.2	0.9
2015-09-24	12.4	9.2	16.8	2.6
2015-09-25	10.8	7.9	15.6	3.3
2015-09-26	7.7	1.6	16.0	1.7
2015-09-27	8.0	0.4	17.7	1.4
2015-09-28	7.9	1.0	16.5	1.1
2015-09-29	9.8	1.5	17.0	2.1
2015-09-30	11.6	4.5	19.6	1.6
2015-10-01	12.6	6.9	15.8	2.9
2015-10-02	11.5	2.4	16.7	2.7
2015-10-03	9.3	1.3	14.8	1.9
2015-10-04	8.8	4.9	15.9	1.4
2015-10-05	8.2	1.9	14.0	2.4
2015-10-06	8.1	5.6	10.2	4.4
2015-10-07	7.2	4.7	10.5	3.9
2015-10-08	7.0	4.9	10.3	2.9
2015-10-09	6.6	4.8	8.5	1.3
2015-10-10	7.4	4.8	10.7	0.9
2015-10-11	7.9	6.2	10.7	1.2
2015-10-12	7.0	6.1	10.6	1.3
2015-10-13	5.0	2.6	10.7	1.3
2015-10-14	0.4	-3.9	7.4	0.9
2015-10-15	1.0	-2.4	8.2	0.6
2015-10-16	1.8	-1.7	4.0	1.7
2015-10-17	1.2	-1.1	8.6	0.8
2015-10-18	2.1	-3.8	12.0	0.6
2015-10-19	0.9	-2.4	5.8	0.7
2015-10-20	4.9	-1.3	7.7	0.7
2015-10-21	7.0	2.8	10.3	2.9
2015-10-22	10.7	6.9	14.5	3.6
2015-10-23	9.1	4.8	14.8	2.0
2015-10-24	6.9	-1.5	11.0	3.0
2015-10-25	8.1	2.9	11.8	3.0
2015-10-26	6.3	1.9	10.4	1.7
2015-10-27	2.4	-1.9	7.7	1.4
2015-10-28	5.3	-0.1	6.8	1.6
2015-10-29	5.1	3.9	6.4	1.5
2015-10-30	5.6	4.6	7.1	1.6
2015-10-31	8.5	5.4	9.8	2.4
2015-11-01	8.7	6.2	10.8	1.9
2015-11-02	7.9	2.2	14.8	2.0
2015-11-03	8.0	2.1	10.0	2.2
2015-11-04	5.2	4.0	9.4	1.7
2015-11-05	4.1	3.0	4.8	1.8
2015-11-06	6.2	4.7	7.1	1.2
2015-11-07	9.8	6.8	13.2	1.9
2015-11-08	6.8	3.6	10.0	2.5
2015-11-09	7.2	3.7	9.3	2.8
2015-11-10	5.2	3.7	9.2	1.9
2015-11-11	1.2	-1.9	7.4	1.3
2015-11-12	3.5	0.1	8.5	1.3
2015-11-13	5.7	-1.9	11.9	2.4
2015-11-14	3.4	0.9	5.9	2.1
2015-11-15	1.7	-2.1	7.3	1.7
2015-11-16	0.4	-4.5	3.2	1.9
2015-11-17	0.9	-3.5	5.8	0.9
2015-11-18	-0.1	-2.3	4.6	0.2

2015-11-19	-1.1	-1.6	0.2	0.0
2015-11-20	-6.0	-8.3	-0.6	0.1
2015-11-21	-6.0	-10.0	-3.5	0.6
2015-11-22	-7.7	-12.0	-1.7	0.8
2015-11-23	-7.4	-10.8	-3.5	0.8
2015-11-24	0.7	-8.5	4.4	1.2
2015-11-25	1.5	0.3	4.9	1.3
2015-11-26	-1.4	-7.1	1.3	0.8
2015-11-27	8.8	0.6	10.1	4.5
2015-11-28	3.7	-1.6	10.1	2.9
2015-11-29	2.9	-1.0	5.5	3.6
2015-11-30	-0.8	-4.2	4.9	1.5
2015-12-01	-1.1	-6.4	5.1	1.9
2015-12-02	0.9	-5.1	5.7	1.3
2015-12-03	1.4	-2.5	6.5	1.8

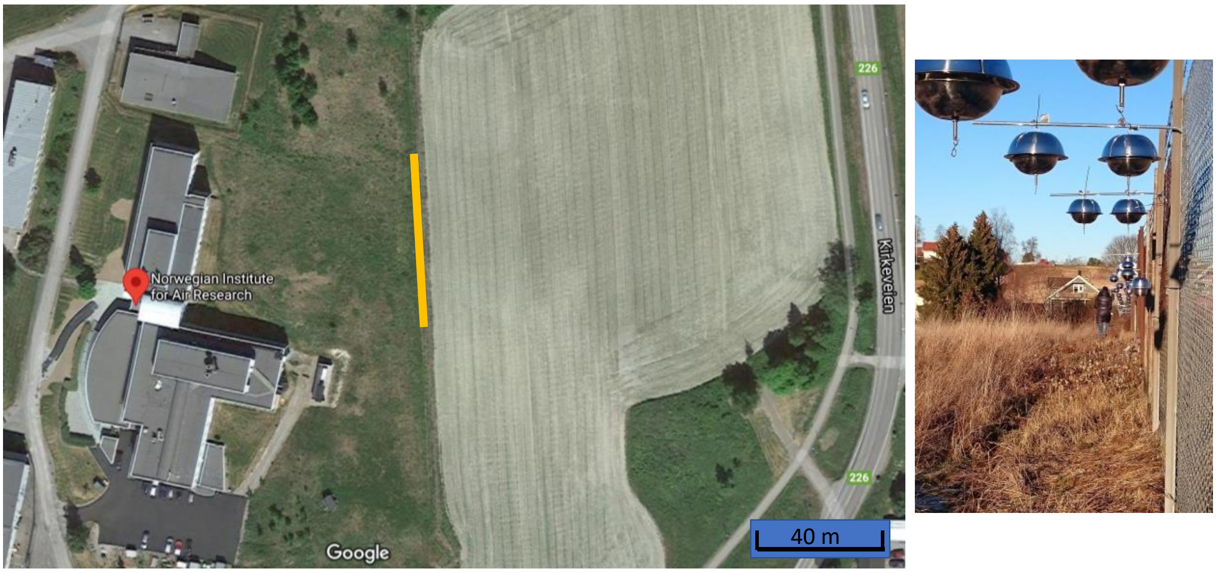

Figure S2: Deployment locations of PUF-PAS, indicated by the orange line.

Table S5: Target compounds according to study phases

	Phase 1	Phases 2 and 3
PAHs	Naphthalene	Naphthalene
	Acenaphthylene	Acenaphthylene
	Acenaphthene	Acenaphthene
	Fluorene	Fluorene
	Phenanthrene	Phenanthrene
	Anthracene	Anthracene
	Fluoranthene	Fluoranthene
	Pyrene	Pyrene
	Benzo(a)anthracene	Benzo(a)anthracene
	Chrysene	Chrysene
	Benzo(b)fluoranthene	Benzo(b)fluoranthene
	Benzo(k)fluoranthene	Benzo(k)fluoranthene
	Benzo(a)pyrene	Benzo(a)pyrene
	Dibenzo(ah)anthracene	Dibenzo(ah)anthracene
	Benzo(ghi)perylene	Benzo(ghi)perylene
	Indeno(123cd)pyrene	Indeno(123cd)pyrene
	Biphenyl	
	Retene	
	Benzo(b)fluorene	
	Benzo-naphtho-thiophene	
	Benzo(ghi)fluoranthene	
	Cyclopenta(cd)pyrene	
	Triphenylene	
	Benzo(j)fluoranthene	
	Benzo(e)pyrene	
	Perylene	
	Dibenzo(ac)anthracene	
	Anthanthrene	
	Coronene	
OCPs	Pentachlorobenzene (PeCB)	
	Hexachlorobenzene (HCB)	Hexachlorobenzene (HCB)
	α-hexachlorocyclohexane (α-HCH)	α-hexachlorocyclohexane (α-HCH)
	β- hexachlorocyclohexane (β-HCH)	β- hexachlorocyclohexane (β-HCH)
	δ- hexachlorocyclohexane (δ-HCH)	γ- hexachlorocyclohexane (γ-HCH)
	p,p'-DDT	p,p'-DDT
	o,p'-DDT	o,p'-DDT
	p,p'-DDE	p,p'-DDE
	o,p'-DDE	o,p'-DDE
	p,p'-DDD	p,p'-DDD
	o,p'-DDD	o,p'-DDD
PCBs	PCB-11	
	PCB-28	PCB-28
	PCB-52	PCB-52
	PCB-101	PCB-101
	PCB-118	PCB-118
	PCB-138	PCB-138
	PCB-153	PCB-153
	PCB-180	PCB-180
PBDEs	BDE-28	BDE-28
	BDE-47	BDE-47
	BDE-66	BDE-66
	BDE-85	BDE-99
	BDE-99	BDE-100
	BDE-100	BDE-138
	BDE-153	BDE-153
	BDE-154	BDE-154
	BDE-183	BDE-183
	BDE-209	BDE-209

Table S6: Percentage recoveries for Phase 1 samples (n=16)

Recovery standard	Minimum	Average	Maximum
D8-naphthalene	83.1	94.9	105
D10-phenanthrene	72.4	80.0	90.0
D12-perylene	79.9	90.2	98.1
PCB 30	50.5	58.5	73.7
PCB 185	69.6	79.2	90.5

Table S7 - Field blank levels and method detection limits for Phase 1.

Compound	Avg. of field blanks (n=3) (ng/sample)	MDL (ng/sample)
Naphthalene	83.3	150
Acenaphthylene	5.46	6.55
Acenaphthene	2.05	2.89
Fluorene	5.65	7.64
Phenanthrene	13.1	20.5
Anthracene	0.423	0.557
Fluoranthene	3.95	6.05
Pyrene	3.15	6.22
Benzo(a)anthracene	0.301	0.180
Chrysene	0.611	1.12
Benzo(b)fluoranthene	0.118	0.118
Benzo(k)fluoranthene	ND	0.440
Benzo(a)pyrene	ND	0.580
Indeno(123cd)pyrene	ND	0.170
Dibenzo(ah)anthracene	ND	0.170
Benzo(ghi)perylene	ND	0.280
Biphenyl	21.1	37.6
Retene	0.868	2.29
Benzo(b)fluorene	ND	0.330
Benzo-naphtho-thiophene	ND	0.270
Benzo(ghi)fluoranthene	0.238	0.398
Cyclopenta(cd)pyrene	ND	0.280
Triphenylene	0.278	0.487
Benzo(j)fluoranthene	ND	0.500
Benzo(e)pyrene	ND	0.800
Perylene	ND	0.490
Dibenzo(ac)anthracene	ND	0.230
Anthanthrene	ND	0.470
Coronene	ND	0.840
Pentachlorobenzene	ND	0.0888
Hexachlorobenzene	ND	0.0178
Alpha-HCH	ND	0.0167
Beta-HCH	ND	0.0311
Gamma-HCH	ND	0.0255
Delta-HCH	ND	0.0389
o,p'-DDT	ND	0.0266
p,p'-DDT	ND	0.0133
o,p'-DDD	ND	0.0167
p,p'-DDD	ND	0.0133
o,p'-DDE	ND	0.00888
p,p'-DDE	ND	0.0144
PCB 11	0.281	0.411
PCB 28	ND	0.00666

PCB 52	ND	0.0144
PCB 101	ND	0.0300
PCB 118	ND	0.0178
PCB 138	ND	0.0178
PCB 153	ND	0.0200
PCB 180	ND	0.0167
PBDE 28	0.00162	0.00607
PBDE 47	0.111	0.160
PBDE 66	0.00180	0.00681
PBDE 85	0.00880	0.0128
PBDE 99	0.147	0.221
PBDE 100	0.0276	0.0384
PBDE 153	0.0140	0.0252
PBDE 154	0.0102	0.0152
PBDE 183	ND	0.00250
PBDE 209	0.0804	0.240

Table S8 – Analytical methods used by participating laboratories

Lab	Sample extraction	Solvent	Clean-up	Instrumentation
A	Soxhlet	Hexane	Acid and silica	GC-MS
B	Buchi B-811 extractor	Dichloromethane	H2SO4 silica column, silica only (for PAHs)	GC-MS
C	ASE	Acetone:petroleum ether (1:1)	No clean-up	GC-MS/MS
D	Soxhlet	Hexane:toluene (9:1), Toluene (PBDEs)	Alox-Florisil, SPE	GC-HRMS
E	Soxhlet	Acetone:hexane (1:1)	Al-silica acid	GC-MS
F	NA			
G	ASE	Acetone:hexane (1:1)	Acid silica + al-oxide	GC-HRMS
H	Soxhlet	Dichloromethane	Silica + H2SO4	GC-MS
J	ASE	Hexane:acetone (3:1)	Na2SO4 column	GC-MS
K	Soxhlet	Hexane:acetone (1:1) – (OCP/PAH)<br>Toluene:acetone (9:1) – (PCB/PBDE)	GPC+silica gel (OCP)<br>Silicagel (PAH)<br>Multilayer silica+Alumina (PCB/PBDE)	GC-uECD (OCP),<br>HPLC/UV-FL (PAH)<br>GG/HRMS (PCB/PBDE)
L	Soxhlet	Hexane:dichloromethane (9:1)	Supelco dioxin prep system-Florisil	GC-HRMS
M	NA			

Table S9 - Phase 1 PAH masses (ng/sampler) flagged if outside of typical sampler variability. Blue cells have low values, yellow cells have high values, determined based on median±(3%uncertainty). ID code numbers refer to the sampler configurations in Table S2 and Fig. 1.*

ID code	1*	2*	3*	4*	5*	6	7	8	9	10	11	12	13	14	15	16	Median	Lower bound	Upper bound
Acenaphthene	37.5	37.6	35.9	37.2	36.3	36.2	35.6	40.2	27.1	43.0	35.4	37.9	22.9	34.5	37.1	37.6	36.7	34.5	38.9
Fluorene	116	111	111	118	108	96.6	106	118	109	111	112	115	69.8	110	119	112	111	99.4	122
Phenanthrene	394	402	403	404	382	319	352	468	404	449	399	432	248	382	408	422	402	375	430
Anthracene	2.23	1.92	1.81	2.04	2.08	1.97	1.43	2.62	1.08	2.02	2.19	2.27	1.12	1.98	1.59	1.84	1.97	1.50	2.44
Benzo(b)fluorene	5.94	5.63	5.28	5.93	5.50	2.65	4.49	4.33	1.12	5.67	5.40	4.67	3.48	5.12	5.72	5.90	5.34	5.09	5.59
Retene	31.9	31.1	31.5	34.9	31.6	18.3	25.1	36.6	19.6	35.1	32.6	34.6	21.3	28.6	31.7	34.1	31.6	28.4	34.9
Fluoranthene	150	149	149	151	145	95.6	118	147	62.3	162	147	155	84.4	134	147	159	147	123	171
Pyrene	75.4	74.8	73.4	79.7	73.3	47.1	61.9	66.6	15.0	83.1	76.8	74.2	45.2	68.0	75.6	82.1	73.8	58.3	89.3
Benzo-naphtho-thiophene	1.30	1.36	1.31	1.52	1.45	0.94	1.18	1.74	0.76	1.63	1.43	1.88	0.72	1.19	1.41	1.58	1.38	0.64	2.13
Benzo(a)anthracene	2.70	2.65	2.49	2.89	2.62	1.49	2.09	3.82	2.07	2.89	2.98	3.46	1.75	2.44	2.55	2.78	2.63	1.39	3.88
Chrysene	8.38	9.63	8.78	8.45	8.04	4.36	7.26	9.50	4.21	11.1	9.28	10.5	5.80	8.70	10.2	10.5	8.74	5.65	11.8
Triphenylene	3.69	4.16	4.19	4.08	3.69	2.59	2.98	6.20	4.37	4.84	4.17	5.72	2.02	3.78	4.33	4.33	4.17	1.82	6.51
Benzo(b)fluoranthene	1.52	2.16	1.71	1.40	1.53	0.83	1.10	2.35	0.91	2.24	1.63	2.25	0.91	1.60	2.05	1.93	1.62	1.10	2.13
Benzo(k)fluoranthene	1.11	1.64	1.18	1.26	1.30	0.73	1.10	2.18	2.07	1.66	1.33	1.81	0.83	1.41	1.68	1.65	1.37	1.17	1.57
Benzo(j)fluoranthene	0.58	1.04	0.95	0.66	0.57	0.25	0.80	1.36	1.43	0.78	0.25	0.99	0.25	0.81	0.83	0.82	0.80	0.68	0.92
Benzo(ghi)fluoranthene	7.00	6.99	6.46	7.77	6.76	3.61	5.46	4.97	1.28	6.93	7.26	6.28	4.12	6.10	6.94	7.19	6.61	5.30	7.93
Benzo(a)pyrene	0.29	0.29	0.29	0.29	0.29	0.29	0.65	1.22	0.29	0.92	0.80	0.86	0.29	0.90	0.78	0.70	0.47	0.37	0.57
Benzo(e)pyrene	2.95	3.85	3.50	3.12	2.98	2.18	3.11	5.00	3.09	4.67	3.49	4.99	1.90	3.27	3.73	3.98	3.38	2.73	4.03
Indeno(123cd)pyrene	1.56	2.40	2.46	2.23	1.81	1.58	1.90	4.55	5.05	3.53	2.28	3.83	1.33	2.34	2.37	2.56	2.35	0.32	4.39
Benzo(ghi)perylene	2.31	3.00	2.84	2.54	2.49	1.72	2.78	5.16	2.74	4.13	2.72	4.89	1.66	2.53	3.21	3.26	2.76	1.78	3.74
Coronene	1.50	1.88	1.79	1.75	1.47	0.98	1.60	3.19	1.39	2.57	1.93	2.83	0.98	1.63	1.92	2.01	1.77	1.20	2.34
SumPAHs	848	854	849	871	819	639	737	935	669	939	850	906	519	801	868	898			

* Samplers 1-5 are identical Tisch samplers

Table S10 - Phase 1 PCB masses (pg/sampler) flagged if outside of typical sampler variability. Blue cells have low values, yellow cells have high values, determined based on median±(3%uncertainty). ID code numbers refer to the sampler configurations in Table S2 and Fig. 1.*

ID code	1*	2*	3*	4*	5*	6	7	8	9	10	11	12	13	14	15	16	Median	Lower bound	Upper bound
PCB 11	3840	4090	1850	3950	1850	8960	1850	5780	7210	4130	4180	4800	7630	3300	4100	3790	4100	0	8670
PCB 28	1220	1090	980	1150	1150	1070	940	1410	1290	1240	1170	1330	760	810	1210	1310	1160	880	1450
PCB 52	810	880	830	870	910	780	650	1090	1040	1030	860	1010	610	670	970	900	875	753	996
PCB 101	1050	890	750	780	790	560	540	1080	900	990	830	1070	420	660	840	890	839	476	1210
PCB 118	290	350	290	320	290	260	250	470	400	390	330	480	200	300	320	370	322	238	406
PCB 153	870	830	770	780	830	620	610	1070	880	1060	890	1280	440	750	850	920	839	715	964
SumPCBs	8080	8130	5470	7850	5820	12200	4840	10900	11720	8840	8260	9970	10060	6490	8290	8180			

* Samplers 1-5 are identical Tisch samplers

Table S11 - Phase 1 OCP masses (pg/sampler) flagged if outside of typical sampler variability. Blue cells have low values, yellow cells have high values, determined based on median±(3%uncertainty). ID code numbers refer to the sampler configurations in Table S2 and Fig. 1.*

ID code	1*	2*	3*	4*	5*	6	7	8	9	10	11	12	13	14	15	16	Median	Lower bound	Upper bound
PeCB	520	730	560	540	630	520	540	520	560	610	510	510	790	530	540	530	540	320	760
HCB	10800	11400	9240	8150	8460	7510	7330	10700	11290	8780	8400	9000	8250	7250	8550	8620	8580	4780	12400
a-HCH	1670	1890	1640	1530	1670	1520	1300	2320	2980	1850	1560	1870	1290	1480	1590	1870	1650	1270	2030
g-HCH	1840	1820	1900	1760	1740	1670	1380	2500	2530	2040	1770	2050	1240	1390	1850	1850	1830	1640	2020
op-DDT	190	230	200	230	200	140	160	280	230	280	220	280	110	170	200	250	210	150	270
pp-DDT	250	280	210	240	250	190	190	350	290	320	250	380	100	160	260	270	250	180	320
pp-DDE	1410	990	940	960	920	730	800	1590	1380	1330	970	1430	590	950	960	1100	970	390	1540
a+g-HCH	3510	3710	3540	3290	3410	3190	2680	4820	5510	3890	3330	3920	2530	2870	3440	3720			
SumDDX	1850	1500	1350	1430	1370	1060	1150	2220	1900	1930	1440	2090	800	1280	1420	1620			

* Samplers 1-5 are identical Tisch samplers

Table S12 - Phase 1 PBDE masses (pg/sampler) flagged if outside of typical sampler variability. Blue cells have low values, yellow cells have high values, determined based on median±(3%uncertainty). ID code numbers refer to the sampler configurations in Table S2 and Fig. 1.*

Number ID	1*	2*	3*	4*	5*	6	7	8	9	10	11	12	13	14	15	16	Median	Lower bound	Upper bound
BDE 28	6.80	7.85	7.40	6.82	7.04	5.79	6.15	9.88	9.28	9.68	7.08	8.88	3.03	6.96	6.88	7.76	7.06	5.74	8.37
BDE 47	62.5	55.2	62.5	55.2	55.2	55.2	55.2	79.5	84.5	92.5	59.5	91.5	55.2	55.2	53.5	53.5	55.2	43.9	66.6
BDE 183	1.25	1.25	4.52	4.68	4.17	2.76	5.81	1.25	12.8	6.58	1.25	8.40	1.25	5.54	5.56	4.87	4.60	0	12.3
BDE 209	120	120	120	120	243	120	663	299	1300	120	120	120	120	221	120	120	120	0	257
SumBDEs	191	184	194	187	309	184	730	390	1407	229	188	229	179	289	186	186			

* Samplers 1-5 are identical Tisch samplers

Table S13 - Assessment of variability in 5 identical Tisch samplers in Phase 1. Values are in ng/sampler for PAHs, and pg/sampler for PCBs, OCPs, and PBDEs.

	Class	Minimum	Maximum	Mean	Std. Deviation	Relative standard deviation (%)
PeCB	OCP	520	730	598	82.4	14%
HCB	OCP	8150	11370	9600	1420	15%
α-HCH	OCP	1530	1890	1680	129	8%
γ-HCH	OCP	1740	1900	1810	62.7	3%
op-DDT	OCP	190	230	209	21	10%
pp-DDT	OCP	210	280	245	22.7	9%
pp-DDE	OCP	920	1410	1050	207	20%
Acenaphthene	PAH	35.9	37.6	36.9	0.73	2%
Fluorene	PAH	108	118	113	3.87	3%
Phenanthrene	PAH	382	404	397	9.17	2%
Anthracene	PAH	1.81	2.23	2.02	0.16	8%
Benzo(b)fluorene	PAH	5.28	5.94	5.66	0.283	5%
Retene	PAH	31.1	34.9	32.2	1.55	5%
Benzo(b)fluoranthene	PAH	1.40	2.16	1.66	0.298	18%
Fluoranthene	PAH	145	151	149	2.33	2%
Pyrene	PAH	73.3	79.7	75.3	2.60	3%
Benzo-naphtho-thiophene	PAH	1.3	1.52	1.39	0.0922	7%
Benzo(a)anthracene	PAH	2.49	2.89	2.67	0.144	5%
Chrysene	PAH	8.04	9.63	8.66	0.606	7%
Triphenylene	PAH	3.69	4.19	3.96	0.253	6%
Benzo(k)fluoranthene	PAH	1.11	1.64	1.3	0.204	16%
Benzo(ghi)fluoranthene	PAH	6.46	7.77	7.00	0.485	7%
Benzo(j)fluoranthene	PAH	0.57	1.04	0.761	0.219	29%
Benzo(e)pyrene	PAH	2.95	3.85	3.28	0.387	12%
Benzo(a)pyrene	PAH	NA (<MDL in all 5 samples)				
Coronene	PAH	1.47	1.88	1.68	0.181	11%
Indeno(123cd)pyrene	PAH	1.56	2.46	2.09	0.392	19%
Benzo(ghi)perylene	PAH	2.31	3.00	2.64	0.282	11%
PCB-11	PCB	1850	4090	3120	1160	37%
PCB-28	PCB	980	1220	1120	91.0	8%
PCB-52	PCB	810	910	861	39.8	5%
PCB-101	PCB	750	1050	852	123	14%
PCB-118	PCB	290	350	310	27.1	9%
PCB-153	PCB	770	870	815	40.3	5%
BDE-28	PBDE	6.8	7.85	7.18	0.445	6%
BDE-47	PBDE	55.2	62.5	58.1	3.97	7%
BDE-183	PBDE	1.25	4.68	3.17	1.77	56%
BDE-209	PBDE	120	243	144	54.8	38%

Table S14: Phase 2 field blanks. PCBs, OCPs, and PBDEs in pg/sample, PAHs in ng/sample. Individual laboratories are distinguished by letter code (A-M).

Lab ID	A	B	C	D	E	F	G	H	J	K	L	M
PCB-28	27.20	115.00	41.00	55.00	95.00	68.88	110.00	7.58		2502.00	79.22	50
PCB-52	44.20	109.00	39.00	40.00	26.00	33.63	76.80	2740.00		1964.00	87.88	2.5
PCB-101	176.00	57.30	13.00	50.00	26.50	47.31	76.20	3483.00		1394.00	38.56	5
PCB-118	48.00	32.80	55.00	60.00	27.00	Nd	42.50	6.09		752.00	11.33	2.5
PCB-138	124.00	43.90	19.00	50.00	23.00	20.07	81.80	12.00		742.00	10.67	5
PCB-153	188.00	60.70	1.00	100.00	23.00	7.64	118.00	13.23		732.00	30.98	5
PCB-180	49.90	4.45	25.00	30.00	40.00	9.45	7.80	17.11		177.00	n.d.	2.5
SumPCBs	657.30	423.15	193.00	385.00	260.50	186.97	513.10	6279.01		8263.00	258.64	72.50
HCB	182	640	450	622.08	NA	203.28	535	18.9	NA	1500	248.8	
α-HCH	30	58.6	146	77.63	22	9.122	1.565	14.25	20216.99	5000	NA	
β-HCH	4.98	ND	32.5	NA	10	10.897	1.565	10252	15359.04	5000	NA	
γ-HCH	43.2	99.7	166	120.51	18	54.939	94.4	30.78	50	5260	NA	
p,p’-DDT	18.6	78.3	40	25	9.5	6.813	126	963	74246.08	3000	7.2	
o,p’-DDT	6.05	43.6	42	35	NA	2.585	69	844	NA	NA	9.85	
p,p’-DDE	47.4	243	98	160.90	31	106.19	29.85	26.95	6106.535	1000	7	
o,p’-DDE	3.915	11.8	84	15	NA	ND	23.45	1351	NA	NA	6.9375	
p,p’-DDD	3.675	1.85	70	35	13	ND	49.05	963	18086.86	1000	10.4	
o,p’-DDD	3.99	1.85	41	15	NA	ND	7.8	8.835	NA	NA	10.6875	
SumHCHs	78.18	158.3	344.5	198.14	50	74.96	97.53	10297.03	35626.03	15260	0	
SumDDX	83.63	380.4	375	285.90	53.5	115.586	305.15	4156.78	98439.47	5000	52.075	
Naphthalene	113.00	92.90	150.34	NA	1102.84	202.82	NA	17.17	223.49	164.00		
Acenaphthylene	1.51	1.83	n,d,	NA	42.04	0.30	NA	n,d	0.75	12.50		
Acenaphthene	7.32	3.08	0.05	NA	30.38	5.00	NA	9.59	8.08	5.00		
Fluorene	11.50	9.18	8.56	NA	95.95	10.08	NA	25.01	12.98	21.50		
Phenanthrene	15.10	23.00	17.25	22.21	288.98	19.12	12.50	39.45	21.48	84.80		
Anthracene	0.20	0.71	1.98	0.00	6.82	0.36	0.07	43.31	1.14	5.00		
Fluoranthene	3.06	12.50	4.75	4.32	46.25	1.18	2.89	25.01	3.52	42.30		

Pyrene	1.67	5.15	4.26	3.51	32.99	15.68	1.99	47.30	2.05	13.70		
Benzo(a)anthracene	0.05	1.10	1.07	0.13	4.03	ND	0.09	<4.5	0.29	5.00		
Chrysene	0.25	1.40	26.45	<1	11.90	0.70	0.03	<4	0.72	5.00		
Benzo(b)fluoranthene	0.12	0.01	0.73	0.25	0.16	0.32	0.17	<6	0.19	5.00		
Benzo(k)fluoranthene	0.08	0.02	0.32	NA	0.17	ND	0.03	27.17	0.25	2.50		
Benzo(a)pyrene	0.08	0.03	1.59	0.08	3.81	ND	0.05	77.12	0.26	2.50		
Dibenzo(ah)anthracene	0.14	0.01	0.18	0.10	2.12	ND	0.03	4.75	0.43	7.50		
Benzo(ghi)perylene	0.12	0.02	0.30	0.08	2.75	0.37	0.05	4.75	0.83	20.00		
Indeno(123-cd)pyrene	0.12	0.01	0.40	0.08	2.21	ND	0.04	2.80	0.73	20.00		
SumPAHs	154	151	218	30.7	1700	260	17.92	323	277	416		
BDE-28	1.71	0.28		5	5	7.8	13.7			246		2.5
BDE-47	18.8	19.2		10.3	12	44.65	19.75			1760		2.5
BDE-66	0.245	0.195		5	NA	NA	16.335			113		2.5
BDE-99	6.32	16.6		10	8	4.69	17.36			776		2.5
BDE-100	1.89	2.99		10	4	4.69	17.275			185		2.5
BDE-138	0.7	NA		15	NA	NA	21.35			6.715		2.5
BDE-153	0.685	0.43		15	8	15.65	20.325			85		2.5
BDE-154	0.57	0.28		15	3	15.65	20			51.8		2.5
BDE-183	1.57	0.55		25	NA	15.65	26.575			31.1		5
BDE-209	248	136.15		500	576	7.8	NA			NA		NA
SumPBDEs	280	177		610	616	117	173			3255		25

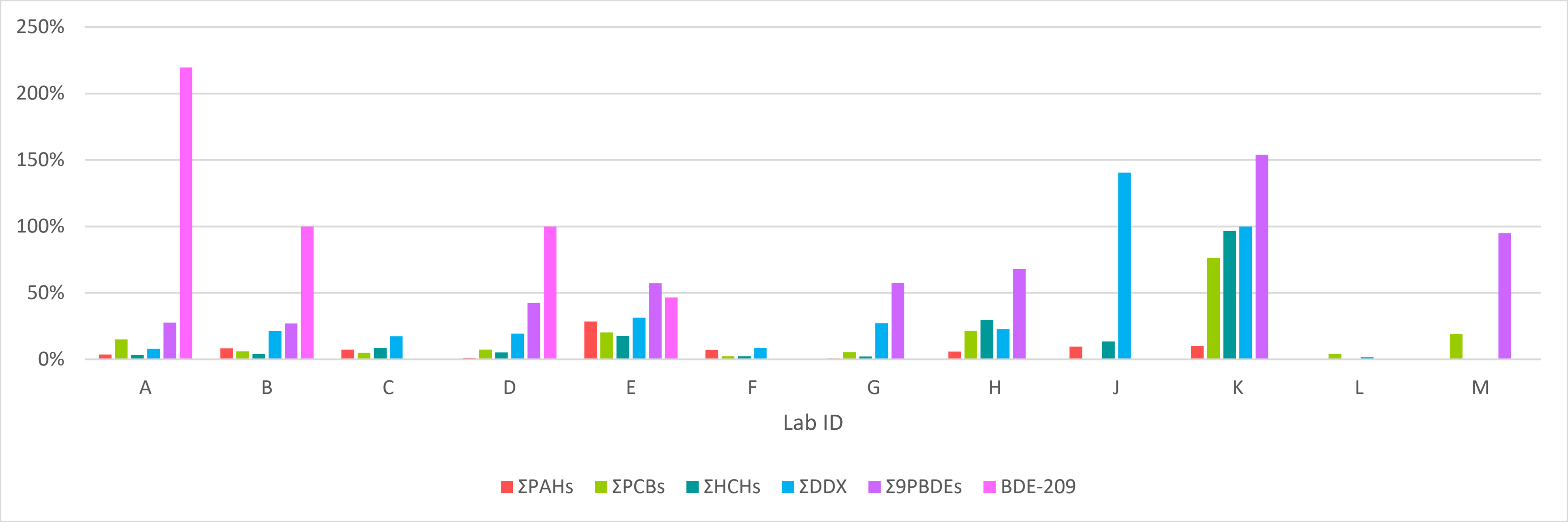

Figure S3: Percentage contribution of field blanks to total sample mass, Phase 2.

*Table S15 - Phase 2 PAH masses (ng/sampler). Grey shading indicates value was below detection limit and is substituted by 0.5*LOD in cases where LOD was available. Individual laboratories are distinguished by letter code (A-M).*

Lab ID	A	B	C	D	E	F	G	H	J	K	L	M	Median
Naphthalene	358	159	241.92	NA	1568.66	401.07	NA	288.891	694.49	374	Not reporting	Not reporting	366
Acenaphthylene	33.9	10.9	<LOD	NA	85.49	28.99	NA	49.063	18.40	45.7			31.4
Acenaphthene	157	50.6	155.1	NA	186.43	133.25	NA	159.421	77.57	154			155
Fluorene	627	166	352.3	NA	560.46	493.38	NA	506.944	274.58	442			468
Phenanthrene	1791	777	1326.8	1716.5	2109.47	1656.52	1680	2239.83	1025.58	1648			1668
Anthracene	80.8	36.6	59.98	71.03	101.95	79.38	131	127.091	45.72	80			79.7
Fluoranthene	611	331	424.51	502.72	591.56	490.02	442	801.801	392.59	887			496
Pyrene	411	237	314.55	381.67	431.16	336.99	316	593.622	276.06	419			359
Benzo(a)anthracene	22.2	13.1	13.57	17.22	26.59	17.84	21.2	83.828	16.41	22			19.5
Chrysene	49.6	23.2	26.4	40.73	127.11	41.43	33.4	120.036	36.55	38.5			39.6

Benzo(b)fluoranthene	14.6	4.79	12.39	18.34	19.37	11.24	18.8	119.91	9.23	17.2			15.9
Benzo(k)fluoranthene	6.1	3.47	<LOD	NA	13.77	10.48	5.26	78.023	8.13	6.27			6.3
Benzo(a)pyrene	4.76	3.22	5.84	4.39	9.06	3.55	5.21	165.541	4.94	7.04			5.1
Dibenzo(ah)anthracene	0.89	0.327	1.00	7.61	13.86	1.53	1.26	4.750	6.17	7.5			3.1
Benzo(ghi)perylene	10.3	5.46	4.91	0.9126	5.69	8.74	9.06	194.165	3.12	20			7.2
Indeno(123-cd)pyrene	6.59	4.16	3.085	5.22	24.18	5.61	6.4	2.8	9.83	20			6.0
SumPAHs	4180	1826	2942	2766	5875	3720	2670	5536	2899	4188			3331

*Table S16 - Phase 2 OCP masses (pg/sampler). Grey shading indicates value was below detection limit and is substituted by 0.5*LOD in cases where LOD was available. Individual laboratories are distinguished by letter code (A-M).*

Lab ID	A	B	C	D	E	F	G	H	J	K	L	M	Median
HCB	13844	21700	20760	24909	NA	11295	22700	30047	NA	14800	13500	Not reporting	20760
α-HCH	1298	2170	2483	2108	149	1617	1730	2275	176855	5000	NA		2139
β-HCH	39	77	33	NA	10	43	155	29839	85842	5000	NA		77
γ-HCH	1078	1850	1460	1706	124	1431	2540	2767	50	5800	NA		1583
p,p'-DDT	146	232	376	217	24	162	126	5133	43991	3000	375		232
o,p'-DDT	128	234	316	230	NA	22	190	938	NA	NA	482		232
p,p'-DDE	719	1190	1164	956	134	1171	726	342	6407	1000	619		956
o,p'-DDE	25	61	70	39	NA	5	23	1494	NA	NA	506		50
p,p'-DDD	10	34	123	35	13	7	49	6285	19671	1000	563		49
o,p'-DDD	9	29	109	42	NA	10	8	4164	NA	NA	647		35
SumHCH	2415	4097	3976	3815	283	3091	4425	34881	262747	15800	NA		4036
SumDDX	1036	1779	2158	1519	171	1378	1122	18356	70069	5000	3194		1779

*Table S17 - Phase 2 PCB masses (pg/sampler). Grey shading indicates value was below detection limit and is substituted by 0.5*LOD in cases where LOD was available. Individual laboratories are distinguished by letter code (A-M).*

Lab ID	A	B	C	D	E	F	G	H	J	K	L	M	Median
PCB-28	1094	2010	820	1474	310	2343	3250	2721	Not reporting	3733	2400	171	2010
PCB-52	1065	1830	946	1185	349	1940	2240	6903		2805	1700	79	1700
PCB-101	892	1330	977	1088	255	1579	1510	6242		1825	1230	50	1230
PCB-118	329	599	331	476	142	654	742	3574		775	476	24	476
PCB-138	368	459	306	428	105	753	586	3636		670	381	21	428
PCB-153	528	648	427	449	93	823	925	3787		818	600	25	600

PCB-180	119	136	21	101	40	231	135	2394		185	24	7	119
SumPCBs	4395	7012	3827	5202	1294	8322	9388	29257		10811	6811	378	6811

*Table S18 - Phase 2 PBDE masses (pg/sampler) by sampler. Grey shading indicates value was below detection limit and is substituted by 0.5*LOD in cases where LOD was available. Individual laboratories are distinguished by letter code (A-M).*

Lab ID	A	B	C	D	E	F	G	H	J	K	L	M	Median
BDE-28	6.3	8.8	Not reporting	23.0	12.0	0.12	7.8	55.0	Not reporting	156	Not reporting	2.5	8.8
BDE-47	71.0	95.0		118.0	23.0	0.16	125.0	17.0		1258		3.8	71.0
BDE-99	24.9	34.5		34.0	18.0	0.05	4.7	27.0		427		2.5	24.9
BDE-100	5.9	7.5		10.0	5.0	0.02	4.7	<LOD		106		2.5	5.0
SumPBDEs	108.1	145.7		185.0	58.0	0.35	142.2	99.0		1947		11.3	108.1

*Table S19: Phase 3 field blanks by Lab ID. PCBs, OCPs, and PBDEs in pg/sample, PAHs in ng/sample. Grey shading indicates value was below detection limit and is substituted by 0.5*LOD in cases where LOD was available. Individual laboratories are distinguished by letter code (A-M).*

	A	B	C	D	E	F	G	H	J	K	L	M
PCB-28	5.05	71.00	38.00	55.00	75.00	75.02	4.69	2171.00		1973.00	13.62	54.464
PCB-52	20.70	57.40	33.50	40.00	26.00	4.54	2.35	2695.00		1181.00	19.54	10.443
PCB-101	154.00	4.80	35.50	50.00	26.50	<LOD	36.50	3463.00		991.00	34.50	5
PCB-118	41.60	3.50	56.00	60.00	27.00	5.28	2.44	6.09		515.00	<LOD	2.5
PCB-138	125.00	4.05	24.00	50.00	23.00	12.52	39.80	12.00		478.00	34.77	5
PCB-153	174.00	6.95	3.00	100.00	23.00	<LOD	63.40	13.23		552.00	100.00	5
PCB-180	42.80	4.45	25.00	30.00	40.00	1.72	7.80	17.11		141.00	40.73	2.5
SumPCBs	563.15	152.15	215.00	385.00	240.50	99.08	156.98	8377.43		5831.00	243.16	84.91
HCB	33.20	579.00	35.25	235.00	NA	103.69	1.57	18.90	NA	1500	14.40	
α-HCH	6.34	61.90	57.25	35.00	20.00	2.50	1.57	14.25	8859.99	5000	NA	
β-HCH	3.30	5.65	NA	NA	10.00	<LOD	1.57	14036.00	13334.39	5000	NA	
γ-HCH	10.10	87.70	38.75	55.00	22.00	16.96	1.57	1335.00	50.00	6.70	NA	
p,p'-DDT	5.00	43.90	30.75	25.00	9.50	0.00	126.00	2700.00	34114.30	3000	7.21	
o,p'-DDT	4.70	2.95	72.25	35.00	NA	3.70	69.00	888.00	NA	NA	9.87	
p,p'-DDE	10.90	190.00	33.75	35.00	27.00	<LOD	29.85	26.95	6073.95	1000	7.02	
o,p'-DDE	3.49	1.60	84.00	15.00	NA	<LOD	23.45	1403.00	NA	NA	6.94	

p,p’-DDD	2.91	1.85	60.50	35.00	13.00	3.33	49.05	2919.00	15233.41	1000	10.42	
o,p’-DDD	3.16	1.85	41.00	15.00	NA	0.00	7.80	8.84	NA	NA	10.69	
SumHCH	19.74	155.25	96.00	90.00	52.00	19.46	4.70	15385.25	22244.37	16.70		
SumDDX	30.16	242.15	322.25	160.00	49.50	7.03	305.15	7945.79	55421.66	5000	52.14	
BDE-28	0.00	0.28		5.00	3.00	<LOD	7.80	13.70		176.00		2.5
BDE-47	0.00	6.63		13.00	8.00	<LOD	122.00	19.75		1439.00		2.5
BDE-66	0.25	0.20		5.00	NA	NA	NA	16.34		87.3		2.5
BDE-99	0.00	6.32		10.00	4.00	<LOD	113.00	17.36		580.00		2.5
BDE-100	0.00	1.73		10.00	1.00	<LOD	4.69	17.28		136.00		2.5
BDE-138	0.70	NA		15.00	NA	NA	NA	21.35		6.72		2.5
BDE-153	0.69	0.43		15.00	2.00	<LOD	15.65	20.33		66.6		2.5
BDE-154	0.57	0.28		15.00	2.00	<LOD	15.65	20.00		40.5		2.5
BDE-183	0.00	0.55		25.00	NA	<LOD	15.65	26.58		25.8		5
BDE-209	0	136.15		500	355	<LOD	7.8	NA		NA		NA
Naphthalene	13.70	98.60	39.45		976.49	77.92		129.13	17.26	91.80		
Acenaphthylene	0.39	1.63	0.22		36.34	0.55		28.81	0.21	12.50		
Acenaphthene	0.58	2.64	27.50		33.04	0.23		18.90	2.10	10.40		
Fluorene	1.39	8.27	3.38		79.12	1.99		50.30	2.20	53.60		
Phenanthrene	3.43	18.00	9.56	7.00	244.83	11.60	1.49	95.12	7.66	407.00		
Anthracene	0.09	0.56	0.33	0.53	7.20	0.40	0.75	43.07	0.31	26.10		
Fluoranthene	1.30	10.20	3.15	2.00	39.65	<LOD	0.53	57.10	1.06	229.00		
Pyrene	0.90	3.71	2.09	1.50	29.18	1.03	0.25	52.00	0.77	73.80		
Benzo(a)anthracene	0.08	0.91	0.60	0.13	3.09	1.14	0.03	61.66	0.14	5.00		
Chrysene	0.47	1.33	1.50	0.50	7.96	0.24	0.03	56.20	0.24	5.00		
Benzo(b)fluoranthene	0.22	0.01	0.35	0.25	0.16	<LOD	0.03	58.60	0.12	5.00		
Benzo(k)fluoranthene	0.04	0.02	0.38	NA	0.17	<LOD	0.03	38.67	0.10	2.50		
Benzo(a)pyrene	0.08	0.03	2.64	0.08	1.90	<LOD	0.05	2.55	0.08	2.50		
Dibenzo(ah)anthracene	0.10	0.01	0.14	0.10	2.53	<LOD	0.03	40.86	0.16	7.50		
Benzo(ghi)perylene	0.19	0.02	0.39	0.08	1.89	<LOD	0.05	4.74	0.21	20.00		
Indeno(123-cd)pyrene	0.08	0.01	0.35	0.08	2.14	<LOD	0.04	2.79	0.36	20.00		
SumPAHs	23.04	145.94	91.98	12.23	1465.68	95.10	3.28	740.48	32.98	971.70		

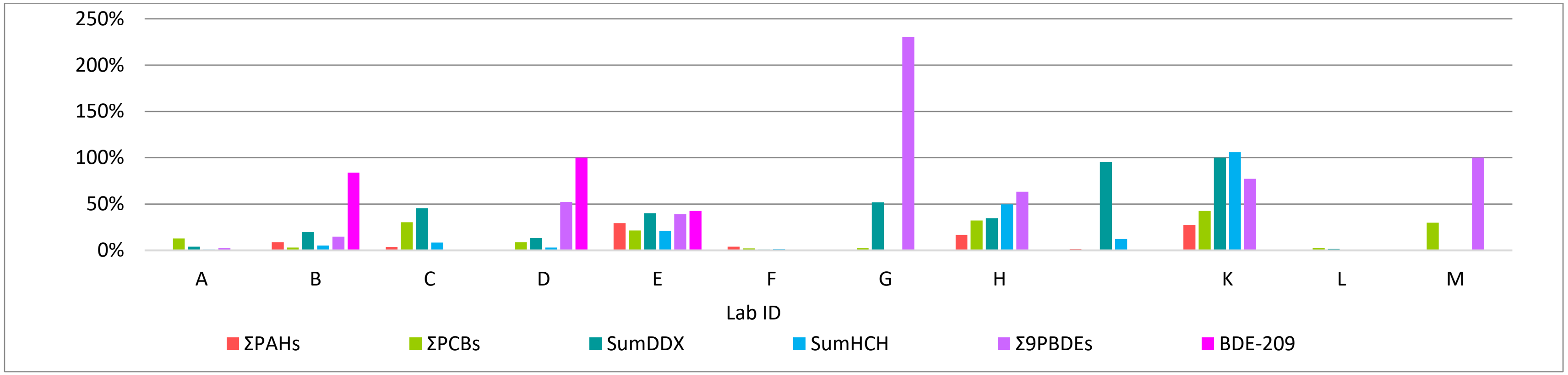

Figure S4: Percentage contribution of field blanks to total sample mass, Phase 3.

*Table S20 - Phase 3 PAH masses (ng/sampler). Grey shading indicates value was below detection limit and is substituted by 0.5*LOD in cases where LOD was available. Individual laboratories are distinguished by letter code (A-M).*

Lab ID	A	B	C	D	E	F	G	H	J	K	L	M	Median
Naphthalene	248	311	127.6	NA	1237.25	238.95	NA	407.68	513.4	249	Not reporting	Not reporting	280.0
Acenaphthylene	33.5	10.9	0.22	NA	64.12	17.64	NA	69.85	18.27	57.3			25.9
Acenaphthene	119	57.7	123.28	NA	116.17	79.42	NA	144.99	52.09	94.6			105.4
Fluorene	547	158	307.11	NA	448.54	304.38	NA	449.95	218.59	351			329.1
Phenanthrene	1668	610	1128.745	1431	1847.08	1036.81	1240	1801.75	885.11	1441			1335.3
Anthracene	74.6	29	33.45	57.98	86.68	49.91	92.3	125.84	41.79	64.2			61.1
Fluoranthene	583	248	458.94	454.46	561.59	307.81	301	643.69	316.2	792			456.7
Pyrene	399	174	330.78	348.828	413.79	218.79	221	489.95	224.55	367			339.8
Benzo(a)anthracene	22	9.86	14.65	17.95	25.32	10.74	15.4	63.06	13.02	20			16.7
Chrysene	47.6	16.5	22.035	42.81	122.3	25.87	25.7	89.97	30.93	37.2			34.1
Benzo(b)fluoranthene	13.8	4.14	13.55	22.96	21	7.14	14.3	81.34	8.89	19			14.1
Benzo(k)fluoranthene	6.47	2.22	3.57	NA	14.96	6.78	4.43	53.26	6.86	6.76			6.8
Benzo(a)pyrene	4.6	2.15	5.265	4.99	7.51	2.61	4.44	2.55	3.87	7.64			4.5
Dibenzo(ah)anthracene	0.63	0.258	0.355	8.237	12.89	0.89	1.08	41.30	5.52	7.5			3.3
Benzo(ghi)perylene	9.73	3.49	5.185	1.105	5.75	<LOD	7.26	4.74	1.83	20			5.0
Indeno(123-cd)pyrene	6.34	2.7	3.53	6.48	22.73	4.02	5.17	2.79	7.14	20			5.8
SumPAHs	3783	1640	2578	2396	5008	2312	1932	4473	2348	3554			2487

*Table S21 - Phase 3 OCP masses (pg/sampler). Grey shading indicates value was below detection limit and is substituted by 0.5*LOD in cases where LOD was available. Individual laboratories are distinguished by letter code (A-M).*

Lab ID	A	B	C	D	E	F	G	H	J	K	L	M	Median
HCB	12161	19600	2910	17879	NA	7339	15400	20473	NA	13000	13700	Not reporting	13700
α-HCH	1091	1510	301	1561	131	946	1190	1031	115196	5000	NA		1141
β-HCH	30	46	NA	NA	10	20	103	25185	66608	5000	NA		75
γ-HCH	924	1320	818	1311	106	887	1120	4747	50	5750	NA		1022
p,p'-DDT	101	149	68	162	26	7	126	3265	29046	3000	464		149
o,p'-DDT	88	151	58	123	NA	14	69	1091	NA	NA	481		105
p,p'-DDE	561	837	341	816	84	726	312	8732	6706	1000	522		726
o,p'-DDE	22	45	89	68	NA	5	23	1402	NA	NA	483		57
p,p'-DDD	5	22	61	35	13	6	49	6342	22422	1000	601		49
o,p'-DDD	11	16	91	15	NA	7	8	2050	NA	NA	630		15
SumHCH	2045	2876	1119	2872	247	1853	2413	30963	181854	16000			2642
SumDDX	787	1220	708	1219	123	764	587	22882	58174	5000	3180		1003

*Table S22 - Phase 3 PCB masses (pg/sampler). Grey shading indicates value was below detection limit and is substituted by 0.5*LOD in cases where LOD was available. Individual laboratories are distinguished by letter code (A-M).*

**Lab ID**	**A**	**B**	**C**	**D**	**E**	**F**	**G**	**H**	**J**	**K**	**L**	**M**	**Median**
PCB-28	1060	1500	155	1235	283	1338	2920	2596	Not reporting	4343	2670	100	1338
PCB-52	1046	1290	154	989	294	1185	1390	5641		3112	1920	61	1185
PCB-101	906	856	149	815	230	904	829	5492		2449	1430	44	856
PCB-118	355	357	70	405	133	390	363	3365		1154	507	24	363
PCB-138	359	333	96	389	70	344	361	3164		1017	630	7	359
PCB-153	533	439	63	419	68	387	461	3325		1167	1020	13	439
PCB-180	115	85	25	110	40	37	44	2320		367	497	35	85
SumPCBs	4374	4860	712	4364	1118	4584	6368	25903		13609	8674	283	4584

*Table S23 - Phase 3 PBDE masses (pg/sampler). Grey shading indicates value was below detection limit and is substituted by 0.5*LOD in cases where LOD was available. Individual laboratories are distinguished by letter code (A-M).*

Lab ID	A	B	C	D	E	F	G	H	J	K	L	M	Median
BDE-28	5.12	5.87	Not reporting	18.3	7	0.07	7.8	46	Not reporting	237	Not reporting	2.5	7.0
BDE-47	61.7	61		87	21	0.14	44.65	42		1861		2.5	44.7
BDE-99	16	22.7		26	11	0.07	23.6	12		745		2.5	16.0
BDE-100	4.93	6.3		10	4	0.02	4.69	19		181		2.5	4.9
BDE-153	0.615	0.43		15	5	0.02	15.65	20		84.3		2.5	5.0
BDE-209	275	162		500	833	0.12	2570	NA		NA		NA	387.5
SumBDEs	363.365	258.3		656.3	881	0.44	2666.39	139.325		3108.3		12.5	363.4
